# Optogenetic induction of hibernation-like state with modified human Opsin4 in mice

**DOI:** 10.1016/j.crmeth.2022.100336

**Published:** 2022-11-14

**Authors:** Tohru M. Takahashi, Arisa Hirano, Takeshi Kanda, Viviane M. Saito, Hiroto Ashitomi, Kazumasa Z. Tanaka, Yasufumi Yokoshiki, Kosaku Masuda, Masashi Yanagisawa, Kaspar E. Vogt, Takashi Tokuda, Takeshi Sakurai

**Affiliations:** 1 Faculty of Medicine, University of Tsukuba, Tsukuba, Japan; 2 International Integrative Institute for Sleep medicine (WPI-IIIS), University of Tsukuba, Tsukuba, Japan; 3 JST PRESTO, Japan; 4 Memory Research Unit, Okinawa Institute of Science and Technology Graduate University (OIST), Okinawa, Japan; 5 Institute of Innovative Research (IIR), Tokyo Institute of Technology, Tokyo, Japan

**Keywords:** optogenetics, OPN4, hibernation, torpor, fiber-less optogenetics, melanopsin, QRFP, body temperature, GPCR, neuroscience

## Abstract

We recently determined that the excitatory manipulation of *Qrfp*-expressing neurons in the preoptic area of the hypothalamus (quiescence-inducing neurons [Q neurons]) induced a hibernation-like hypothermic/hypometabolic state (QIH) in mice. To control the QIH with a higher time resolution, we develop an optogenetic method using modified human opsin4 (OPN4; also known as melanopsin), a G protein-coupled-receptor-type blue-light photoreceptor. C-terminally truncated OPN4 (OPN4dC) stably and reproducibly induces QIH for at least 24 h by illumination with low-power light (3 μW, 473 nm laser) with high temporal resolution. The high sensitivity of OPN4dC allows us to transcranially stimulate Q neurons with blue-light-emitting diodes and non-invasively induce the QIH. OPN4dC-mediated QIH recapitulates the kinetics of the physiological changes observed in natural hibernation, revealing that Q neurons concurrently contribute to thermoregulation and cardiovascular function. This optogenetic method may facilitate identification of the neural mechanisms underlying long-term dormancy states such as sleep, daily torpor, and hibernation.

## Highlights

• We developed a GPCR(Gq)-based optogenetic tool using modified human OPN4 (OPN4dC)
• OPN4dC optogenetics exhibited a high degree of sustainability and reproducibility
• OPN4dC is useful for 24 h neuronal excitation and non-invasive stimulation
• OPN4dC induces a hibernation-like state similar to natural hibernation

## Motivation

Optogenetics is used to manipulate neurons at a high temporal resolution to understand the neuronal mechanisms of many physiologies. Increasing evidence suggests that current tools are effective for short-term manipulation (seconds to minutes); however, the effects of manipulating neurons are difficult to examine for a longer period (hours to days) on long-term behavioral and physiological responses, such as hibernation. This study aimed to establish a method that allows stable and long-lasting (∼24 h) neuronal manipulation with high reproducibility using a modified OPN4 (melanopsin), a highly photosensitive G protein-coupled-receptor-type photosensor.

## Graphical abstract

**Figure:**
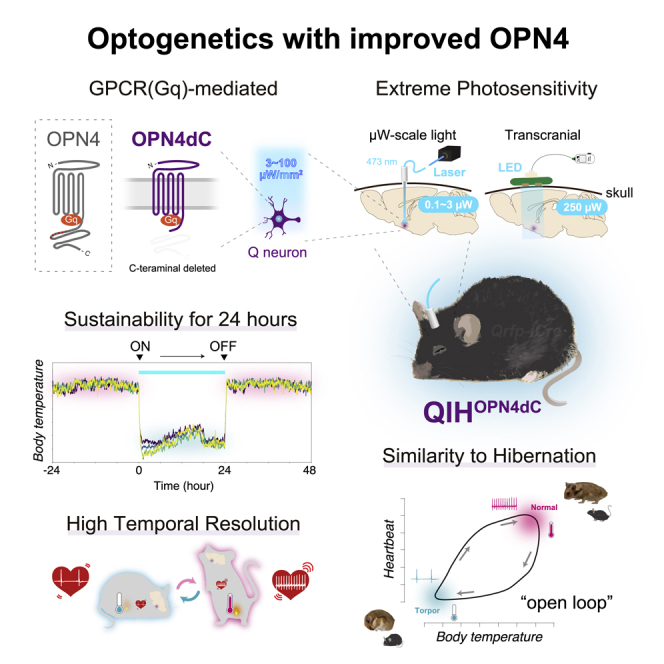

## Teaser

To control the induced hibernation-like state with higher time resolution in mice, Takahashi et al. develop an optogenetic method using human opsin4 (OPN4), a G protein-coupled-receptor-type blue-light photoreceptor. Modified OPN4 stably induces hypothermia for 24 h via extremely low-power light illumination.

## Introduction

Recent advances in neuronal manipulation techniques with appropriate operating periods and temporal resolutions have enabled the study of the effects of the excitation or inhibition of particular neuronal circuits on behavior. Optogenetics has helped elucidate the consequences of manipulation of particular neurons *in vivo* at a high spatiotemporal resolution, providing important insights in neuroscience.^1^^,^^2^ Channel-rhodopsin2 (ChR2) is the most commonly used opsin in optogenetics and evokes action potentials in neurons using brief pulses of blue-light illumination.^3^^,^^4^ Since the first published report of applying ChR2 for optogenetics, various modified ChR2s have been developed to improve the sensitivity (e.g., ChR2/H134^5^^,^^6^) and alter the absorbance spectrum (e.g., crimson,^7^ ChRmine^8^^,^^9^). ChR2 and its variants are conventionally used for relatively short-term (seconds to minutes) manipulations, and it is challenging to examine their long-term effects (hours to days) on physiological changes of slow processes, such as circadian rhythms,^10^ sleep,^11^ and torpor^12^ with optogenetics. Stabilized step function opsin (SSFO), a bistable excitatory variant of ChR, sustains an open state for 30 min by a single brief light pulse;^13^ however, the excitatory manipulation of neurons for over several hours remains challenging. Most neuropeptide and neuromodulator receptors, such as monoamines and acetylcholine, are G protein-coupled receptors (GPCRs),^14^ which evoke slow modulations of neuronal activity, such as changes in membrane potential and input resistance, compared with ion channel receptors. Therefore, a tool for light-inducible G protein signaling would be valuable for examining the functions governed by metabotropic neurotransmission.

Hibernation serves as a biological strategy for thermostatic mammals to adapt to harsh environments, typically described as regulated, hypothermic, and hypometabolic states that last longer than 24 h.^12^^,^^15^^,^^16^^,^^17^ We recently identified a neuronal population that induces a hibernation-like hypometabolic state in mice and rats,^18^ which normally do not hibernate.^19^ These neurons, called quiescence-inducing neurons (Q neurons), express a gene encoding a neuropeptide, pyroglutamylated RFamide peptide (QRFP), and reside in the anteroventral periventricular nucleus (AVPe) in the preoptic area (POA) of the hypothalamus,^18^ known as the thermoregulatory region, which regulates the thermogenic capacity of brown adipose tissue (BAT) via the sympathetic nervous system in mice.^20^ The induced long-lasting (for several days) hypothermic/hypometabolic state, which we termed the Q-neuron-induced hypothermic/hypometabolic state (QIH), was similar to hibernation, including resetting of the body temperature set point.^18^ To induce QIH, we used chemogenetics, a stimulatory hM3Dq designer receptor exclusively activated by the designer drug (DREADD) system.^21^ The mouse core body temperature (*T*_*B*_) and metabolic rate dropped immediately after clozapine-N-oxide (CNO) administration, while mice recovered from hM3Dq DREADD-mediated QIH (hereafter, QIH^M3Dq^) slowly, requiring several days to fully recuperate.^18^ From this perspective, the kinetics of *T*_*B*_ changes in QIH^M3Dq^ do not recapitulate those observed in natural hibernation, especially in terms of return from the hypometabolic state.^12^^,^^22^ This slow recovery process hinders the examination of the effects of QIH-related hypometabolism on various physiological processes, during which the effects of QIH on various physiological functions may be obscured. Therefore, we attempted to develop a technique that would allow the excitatory manipulation of specific neurons over a long period (∼24 h) with high temporal resolution.

The opsin family of proteins is a GPCR-type photoreceptor found in animals.^23^ Among them, opsin4 (OPN4; also known as melanopsin) is expressed only in a small subset of retinal ganglion cells, intrinsically photosensitive retinal ganglion cells (ipRGCs), in primates and rodents.^24^^,^^25^ OPN4 mainly couples to the Gq subclass of G proteins and responds to blue light, playing an important role in non-visual photoreception in mammals, such as the pupillary light reflex and light entrainment of the circadian clock.^24^^,^^26^ Exogenously expressed human OPN4 induces a transient current evoked by blue light in Purkinje cells, which is turned off by yellow light,^27^ suggesting its usefulness as an optogenetic tool. A previous study showed that orexin-producing neurons were excited by hOPN4 with short-term (5 s) photostimulation, resulting in the induction of wakefulness in mice.^28^ Although OPN4 appears to be an ideal tool for long-term neural manipulation based on its properties, neural manipulation for more than several hours has not been reported.

Herein, we provide evidence that optogenetics with modified OPN4 is useful for inducing and maintaining long-term (up to 24 h) QIH with rapid recovery in mice, which is quite similar to hibernation. C-terminally truncated OPN4 (OPN4dC) was activated by low-power light, allowing us to induce QIH with very low light intensity, which even enables non-invasive transcranial stimulation with blue-light-emitting diodes (LED). This study shows that GPCR-type optogenetic manipulation of neurons is a powerful technique for examining neural function in slow and sustained physiological processes (hours to one day) with a high spatiotemporal resolution.

## Results

### OPN4dC optogenetics evokes hibernation-like state with high time resolution

**Figure 1:**
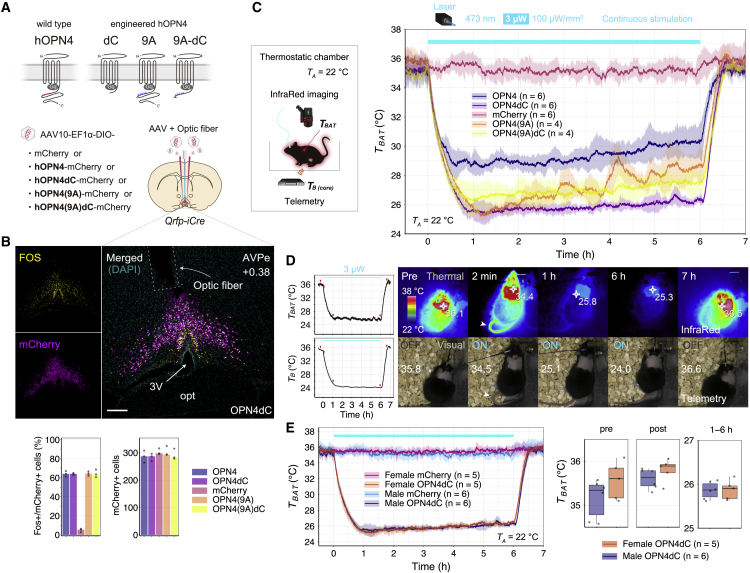
OPN4-mediated optogenetics for long-term neuronal excitation *in vivo* (A) Schematic diagram showing Q neurons specific manipulation by using Cre-activatable (double-floxed inverted open reading frame [DIO]) AAV. AAV carrying hOPN4-, hOPN4dC-, hOPN4(9A)- or hOPN4(9A)dC-mCherry, respectively, was bilaterally injected into the AVPe in *Qrfp-iCre* mice. Q-mCherry mice expressing mCherry in Q neurons were used for the control group. Optic fibers were unilaterally implanted above the AVPe. The images representing each OPN4 structure were modified from a figure in a previous report.^32^ AVPe, anteroventral periventricular nucleus. (B) Top, representative images of coronal brain sections (AP + 0.38 mm from bregma) showing expression of OPN4dC-mCherry (magenta) and c-Fos (yellow) 90 min after light stimulation of OPN4dC-expressing Q neurons and insertion tract of an optic fiber. 3V, third ventricle; opt, optic tract. Scale bars, 200 μm. Bottom, percentages of c-Fos-positive neurons to total mCherry-positive neurons and total numbers of mCherry-positive neuron. Data are shown as means with individual plots (n = 3 mice). (C) The effect of blue-light exposure (473 nm, 3 μW at the fiber tip, 100 μW mm^−2^, 6 h) to the OPN4s on mice body temperature (n = 4–6 mice/group). Start time of light exposure was set to time 0. *T*_*BAT*_ and abdominal core body temperature (*T*_*B*_) were monitored simultaneously using the thermographic camera and telemetric system. Ambient temperature (*T*_*A*_) was set at 22°C using temperature-controlled thermostatic chamber. (D) Left, representative traces of *T*_*BAT*_ and *T*_*B*_ during QIH^OPN4dC^. The arrowheads indicate time points corresponding to images shown in right. Right, representative images of QIH^OPN4dC^ mice expressing OPN4dC-mCherry obtained via thermographic and visual cameras. The figures indicate *T*_*BAT*_ (top) and *T*_*B*_ (bottom) at each time point. The arrow heads indicate tail and heat dissipation 2 min after start of laser stimulation. QIH was induced by blue light (3 μW) from time 0. See also Video S1. (E) Comparison of effects of QIH^OPN4dC^ on *T*_*BAT*_ in females (n = 5 mice) and males. Male data are same as those presented in (B). Left, entire *T*_*BAT*_ trace. Right, average values of *T*_*BAT*_ of Q-OPN4dC group (pre, −0.5–0 h; post, 6.5–7 h, QIH, 1–6 h). The lines and shading in the graphs in (C) and (E) denote the mean and SD of each group, respectively. See also Figures S1–S3.

To manipulate Q neurons with a higher time resolution, we established optogenetics with modified human OPN4 to induce long-term QIH in mice. The C-terminal region of mammalian OPN4 contains a cluster of phosphorylation sites required for arrestin binding, leading to OPN4 inactivation.^29^^,^^30^ Thus, the truncation of this region or the introduction of mutations at the phosphorylation sites in the C-terminal tail of OPN4 may enhance signal transduction. We tested OPN4dCs, OPN4 with mutations at nine phosphorylation sites (OPN4(9A)), and OPN4 with mutations at the phosphorylation sites and a deletion of the subsequent C-terminal region (OPN4(9A)dC) for optogenetic induction of QIH (Figure 1A). To target Q neurons, Cre-dependent adeno-associated viral (AAV) vectors carrying *Opn4* and its variants were injected into the AVPe of *Qrfp-iCre* knockin mice.^18^ After continuous illumination by a 473 nm blue laser with optic fiber (3 μW light power at the fiber tip, 100 μW mm^−2^ irradiance, 6 h stimulation period) in the AVPe, the number of c-Fos-positive neurons increased in OPN4-expressing neurons, showing that Q neurons were efficiently excited (Figure 1B). Under the same conditions, we measured the surface temperature in the interscapular area in which BAT is located (hereafter referred to temperature around BAT [*T*_*BAT*_]) using infrared thermal cameras. The photostimulation of Q neurons with OPN4dC triggered eminent drops of *T*_*BAT*_, and hypothermia was stably sustained for 6 h in mice (Figure 1C). Notably, any form of OPN4s induced QIH efficiently and continuously while shining light with power of 3 μW (100 μW mm^−2^), which was much lower than that usually used for other optogenetic methods (3–30 mW).^31^ The C-terminal deleted form, OPN4dC, lacking all phosphorylation sites, was the most effective, while the amounts of OPN4 expression in cells were compatible with each variant (Figures 1C and S1). Thus, we used OPN4dC fused with mCherry in subsequent experiments and referred to the OPN4dC-mediated QIH as QIH^OPN4dC^.

*Qrfp-iCre* mice expressing OPN4dC-mCherry in the AVPe (Q-OPN4dC mice) showed immobility, rapid decline in body temperature, and vasodilation in tails 2 min after the initiation of light illumination (Figure 1D), which is similar to QIH^M3Dq^. However, contrary to the slow recovery observed in QIH^M3Dq^, *T*_*BAT*_ and *T*_*B*_ increased rapidly immediately after light termination in QIH^OPN4dC^, taking approximately 30 min to reach normal levels (Figures 1C and 1D; Video S1). We confirmed that female mice also showed a similar sufficient and persistent hypothermia using the same method (Figure 1E) because sex differences exist in fasting-induced daily torpor and that the duration of torpor is longer in female mice.^33^^,^^34^ The optogenetic induction of QIH may help identify the physiological changes in the recovery/arousal phase of hibernation and the mechanisms underlying sex differences.

Video S1. Representative thermal and visual imaging of QIHOPN4dC mouse, related to Figure 1 Light stimulation was applied to *Qrfp-iCre* mouse expressing hOPN4dC-mCherry in the AVPe via an optic fiber (3 μW from the fiber tip, 473-nm, 6 h from time 0). Heart rate and core body temperature are also simultaneously taken by telemetry sensors. Video plays at 60x speed. Images in Figure 1D are created from this video.

### Activation of OPN4dC leads to membrsane depolarization and calcium mobilization in neurons

We performed *in vitro* studies for the protein characterization of OPN4dC to indicate that blue-light illumination evoked intracellular signaling that excites neurons. First, the membrane localization of OPN4dC-mCherry and wild-type OPN4-mCherry was confirmed in HEK293T cells (Figure S2A). Since mammalian OPN4 mainly activates Gq signaling,^35^ we measured the concentration of inositol 1-phosphate (IP1), which is a metabolite in the Gq-signaling cascade.^36^ The IP1 assay revealed that continuous blue-light illumination (1 μW/mm^2^ for 20 or 30 min) increased the amount of IP1 in cells expressing either wild-type OPN4 or OPN4dC and that OPN4dC had a higher effect than that of the wild type (Figure S2B). Cells expressing hM3Dq treated with CNO for 20 min generated much higher amounts of IP1 than OPN4s (Figure S2B). We also confirmed the induction of Ca^2+^ transients using blue light. In either the assay system using fluorescence-based Ca^2+^ indicator (Fluo4-AM) or bioluminescence-based indicator (Nano-lantern), OPN4dC exhibited a higher Ca^2+^ response to light than that of wild-type OPN4, as expected (Figures S2C and S2D). Next, luciferase (Luc) activity driven by the nuclear factor of activated T cell (NFAT) response element was used to examine the longer effects of blue light on OPN4-mediated Gq signaling.^37^ LED light illumination (1 μW/mm^2^ for 20 min) increased Luc activity, which was sustained for a longer time in cells expressing OPN4dC compared with those expressing wild-type OPN4 (Figure S2E), while the protein expression levels did not significantly differ between them. OPN4dC was sensitive in this assay (half-maximal effective concentration [EC50]; 9.95 nW/mm^2^) for blue LED light stimulation, and 475 nm blue light efficiently induced the cellular response, compared with the other wavelengths of light with the same power (405 nm violet and 520 nm green) (Figures S2F and S2G), which was consistent with previous studies using full-length OPN4.^24^^,^^29^

Ectopic expression of wild-type hOPN4 demonstrably increases the firing rate of orexin-producing neurons, which is a relatively slow and long-lasting response.^28^ We confirmed that OPN4dC induced action potentials in primary cortical neurons in culture by whole-cell patch-clamp recording using a 20 s blue-light stimulus (Figure S3A). Exposure to blue light (120 μW at the fiber tip, 150 μW/mm^2^, 20 s) depolarized the membrane potential and increased the firing rate (Figures S3A–S3C). The latency to the first action potential firing from the start of light illumination was 6.09 ± 3.83 s (n = 7 neurons from three independent preparations), which is longer than channel-type optogenetic proteins (e.g., ChR2^11^). Moreover, the increase in firing rate and membrane potential depolarization was sustained even after the termination of light stimulation under this condition, while the duration varied among neurons (Figures S3B and S3C). We then performed *in vivo* electrophysiology recordings in *Calb1*-positive pyramidal neurons of the hippocampal CA1^38^ in freely moving mice and showed that their firing frequency was increased during non-invasive stimulation (10 mW at the fiber tip positioned on the brain surface, 10 s, 10 times) (Figure S3D). In this case, the firing frequency returned to the basal level within 15 s after stimulation. Neurons expressing OPN4dC persistently showed a light response during repeated stimulation (Figure S3E).

We then confirmed whether Q neurons expressing OPN4dC in the slice preparations responded to light stimulation by Ca^2+^ imaging with GCaMP6s.^39^ The intracellular Ca^2+^ concentration in Q neurons was clearly increased during stimulation (75 μW at the fiber tip positioned above the neurons, 3 s, 10 times) and returned to the baseline immediately after the termination of photostimulation (Figure S3F). These results indicate that the C-terminal deletion of OPN4 enhances the intracellular response and that OPN4dC has an obvious excitatory effect on neurons.

### *In vivo* characterization of hOPN4dC

**Figure 2:**
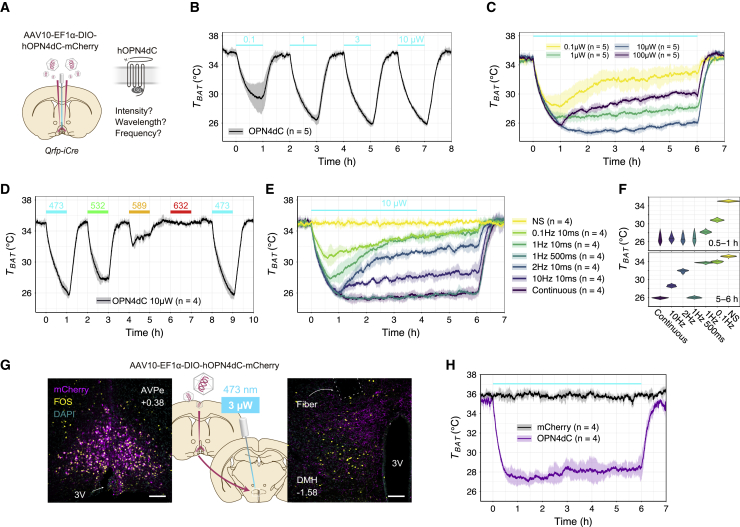
Optimization of OPN4dC light stimulation (A) Schema depicting the position of the AAV10-EF1α-DIO-hOPN4dC-mCherry injection site and optic fiber relative to the targeted neural population (Q neurons, magenta) in the AVPe of *Qrfp-iCre* mice. (B) *T*_*BAT*_ fluctuations in a series of light stimulation of OPN4dC at various intensities (473 nm, 0.1, 1, 3, and 10 μW at the fiber tip, 1 h). Photostimulation was sequentially repeated at an interval of 1 h (n = 5 mice). (C) Average traces of *T*_*BAT*_ for light intensity verification using weaker light (0.1, 1, and 10 μW, 6 h) and stronger light (100 μW, 6 h). The same mice were used for the four groups (n = 5 mice, same animals used in B). (D) Wavelength dependency in OPN4dC-induced *T*_*BAT*_ changes in Q-OPN4dC mice with optical stimulation (n = 4 mice). The colored lines indicate the period of optical stimulation. The power of light for stimulation was 10 μW in all conditions (473, 532, 589, and 632 nm). (E) Frequency dependency in OPN4dC-induced *T*_*BAT*_ changes in Q-OPN4dC mice with 10 μW optical stimulation (n = 4/group from four mice). For pulse-stimulation group, 10-ms-width light pulse in the indicated frequency was used. NS, no stimulation; continuous, continuous stimulation. (F) Violin plots of *T*_*BAT*_ at 0.5–1 and 5–6 h from the beginning of laser stimulation. (G) Schematic diagram of the investigated neural circuit. Q neurons in the AVPe were unilaterally transduced with AAV-DIO-OPN4dC, and optic cannulas were unilaterally implanted at the DMH. Axons of Q neurons at the DMH were manipulated by blue light (3 μW, 6 h). Representative tissue images show the expressions of OPN4dC-mCherry (magenta), c-Fos (yellow), and nuclei stained with DAPI (cyan) in the AVPe and the DMH. Scale bars, 100 μm. DMH, dorsomedial hypothalamus. (H) Changes in *T*_*BAT*_ of Q-OPN4dC mice after photoactivation of axons of Q neurons at the DMH (n = 4 mice/group). Lines and shading in (B)–(E) and (H) denote mean and SD of each group, respectively. See also Figure S4.

Next, we optimized the stimulation condition of OPN4dC at the behavioral level (Figure 2A). To assess the effect of light intensity on QIH, we applied various laser powers (0.1, 1, 3, or 10 μW at the fiber tips) in sequence to induce QIH^OPN4dC^ (Figure 2B). All conditions we tested induced marked hypothermia, but 0.1 μW (3.2 μW mm^−2^) light had a relatively weak effect. These data indicate that OPN4dC expressed in Q neurons responds to extremely weak light (as low as 0.1 μW) and that the effect is saturated at 3 μW (Figure 2B). However, the hypothermia did not persist at a higher light intensity (100 μW at the fiber tips), although the laser power was lower than that normally used (Figure 2C). In a previous study, we succeeded in inducing short-term hypothermia by SSFO with moderate light intensity (10 mW, 1 s width, every 30 min),^18^ whereas the light intensity suitable for OPN4dC was insufficient for either SSFO or ChR2 under all tested conditions (Figure S4). This indicates that OPN4dC and other opsins are efficiently activated under specific light conditions to be optimized according to their properties.

The peak of spectral sensitivity of mammalian OPN4 is approximately 480 nm, which lies in the blue/cyan range of visible light.^24^ We next examined the dependence of wavelength on the stimulation of OPN4dC through QIH induction (10 μW at the fiber tip, 1 h; Figure 2D). Activation of OPN4dC using blue (473 nm) or green (532 nm) lasers triggered a remarkable drop in *T*_*BAT*_, whereas the effect of yellow light (589 nm) was weaker. We did not observe any effect of red light (632 nm) on *T*_*BAT*_, suggesting that OPN4dC could be used in combination with other optogenetic tools (e.g., red calcium indicator). This wavelength dependency of OPN4dC activity is consistent with a previous report on full-length OPN4^24^ and our *in vitro* study (Figure S2G).

To determine whether the decrease in body temperature depended on the frequency and duration of each light pulse, we applied light illumination to Q neurons under various stimulation conditions (10 μW at the fiber tip, 6 h). Photostimulation at 0.1, 1, 2, and 10 Hz decreased *T*_*BAT*_ in a frequency-dependent manner in the early phase (0.5–1 h) (Figure 2E). However, the 10 ms pulse conditions were insufficient to sustain hypothermia during the stimulation (Figure 2F). Among these conditions, only 1 Hz, 500-ms-wide light pulses and continuous stimulation succeeded the long-lasting QIH, suggesting that appropriate amounts of photons are necessary for OPN4dC activation (Figure 2E).

### OPN4dC-mediated axonal fiber excitation

We previously reported that the dorsomedial hypothalamus (DMH), which is implicated in thermoregulation,^40^ is the main target of Q neurons.^18^ Since OPN4dC was expressed in the axons of Q neurons in the DMH, we attempted optogenetic activation of OPN4dC in the axonal fibers to induce behavioral changes. We implanted optic fibers in the DMH unilaterally in Q-OPN4dC mice and performed axonal photostimulation (3 μW at the fiber tip, 6 h; Figure 2G). Although the effect on body temperature was slightly weaker than that on cell bodies, unilateral axonal stimulation in the DMH effectively induced *T*_*BAT*_ reduction and c-Fos expression (Figure 2H). This result indicates that OPN4dC is also useful for examining neuronal functions at the circuit level.

### OPN4dC optogenetics maintains hypothermia for 24 h with good reproducibility

**Figure 3:**
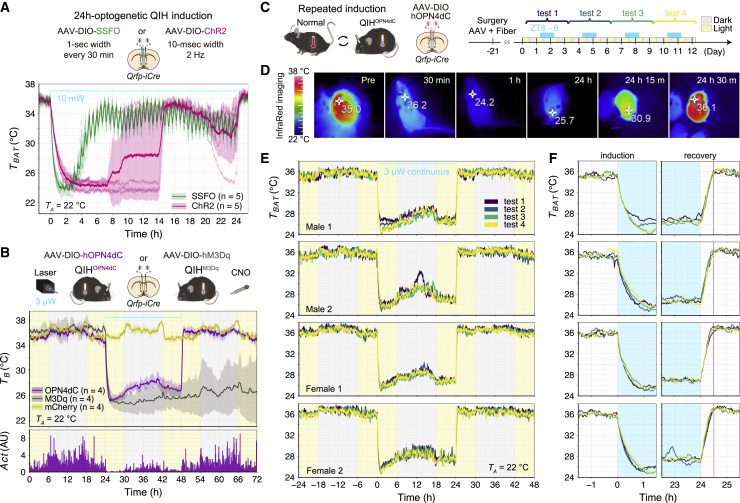
OPN4dC optogenetics permits 24 h behavioral change with reproducibility (A) 24 h QIH induction using ChR2(H134R) and SSFO. ChR2 and SSFO virally expressed in the AVPe were stimulated by blue light (ChR2; 10 mW, 2 Hz, 10 ms width, 24 h, SSFO; 10 mW, 1 s width, every 30 min for 24 h) (n = 5 mice/group). (B) Comparison of QIH^OPN4dC^ with QIH^M3Dq^ (n = 4 mice/group). *T*_*B*_ was measured by a telemetry transmitter, which was implanted interperitoneally. QIH^OPN4dC^ was induced by continuous light (3 μW, 24 h). In QIH^M3Dq^, mice expressing hM3Dq in the AVPe were injected by CNO intraperitoneally (1 mg/kg). Locomotor activity (*Act*) of Q-OPN4dC was plotted in the bottom panel. AU, arbitrary unit. (C) Schema of experimental procedure of repeated 24 h QIH^OPN4dC^ (n = 4 mice). 24 h stimulation was repeated four times at 2 day intervals. The yellow and gray backgrounds indicate 12 h periods of light and darkness, respectively. Laser illumination was initiated at ZT6 (2 p.m.). (D) Representative thermographic images of a Q-OPN4dC mouse. (E) *T*_*BAT*_ were recorded in the repeated 24 h QIH^OPN4dC^ from four independent mice. QIH induced by continuous stimulation (3 μW, 24 h) of OPN4dC was repeated four times at 2 day intervals. (F) Magnified view of (E) plotting *T*_*BAT*_ in the induction and recovery period corresponding to each mouse. The cyan-shaded region marks the light delivery period. Red line shows 30 min after stimulation offset. The lines and shading in (A) and (B) denote the mean and SD of each group, respectively. See also Figure S5.

We next attempted to optogenetically induce a 24-h-long QIH. First, we used the commonly used opsins ChR2 and SSFO. For the use of ChR2, we tested various stimulating frequencies and found that 2 Hz was the most effective for the short-term induction of QIH. Both opsins induced a profound hypothermic phenotype, which persisted for approximately 10 h with ChR2 and 3 h with SSFO. However, we failed to maintain QIH expression for 24 h using these opsins (Figure 3A). In contrast, OPN4dC was able to induce a 24-h-long QIH with weak light (3 μW at the fiber tip, 24 h; Figure 3B). Even though the mice maintained a hypothermic state during the stimulation, we observed a small increase in locomotor activity (*Act*) in the dark period, which correlated with *T*_*B*_ (Figure 3B, bottom). This increase was reproducible in each attempt with the same time course, suggesting the possibility that, even during the long robust hypothermia, a circadian influence remained and that mice became active in the dark period. The *T*_*B*_ change during QIH^OPN4dC^ was similar to that of QIH^hM3Dq^, especially during the induction phase (Figure 3B). However, QIH^OPN4dC^ exhibited rapid recovery from hypothermia after lights off, which is in stark contrast to QIH^hM3Dq^. We repeated 24 h-long stimulation four times at an interval of 2 days (3 μW, 24 h per stimulation) to examine the reproducibility of QIH^OPN4dC^ (Figure 3C). Q-OPN4dC mice (male, n = 2; female, n = 2) showed stable QIH every time, with small variations among individuals and trials (Figures 3D and 3E). The body temperature in all individuals returned to normal within 30 min of the end of the laser stimulation (Figure 3F). The good reproducibility within the same mouse suggested that continuous laser stimulation for as long as 24 h did not cause significant tissue/cell damage or bleaching of OPN4dC. μW-scale photostimulation, which is suitable for OPN4dC optogenetics, did not induce the expression of tissue inflammation and injury markers, glial fibrillary acidic protein (GFAP), and ionized calcium-binding adaptor molecule 1 (Iba1) (Figure S5). In contrast, stimulation with 10 mW at 2 Hz caused a significant increase in Iba1 and GFAP immunoreactivity (Figures S5C–S5E), suggesting that ChR2 may fail to sustain QIH due to local brain tissue damage. Collectively, these results indicate that OPN4dC optogenetics in Q neurons works without causing tissue damage and with extreme sensitivity, sustainability, and reproducibility.

### Fiberless transcranial manipulation of Q neurons

**Figure 4:**
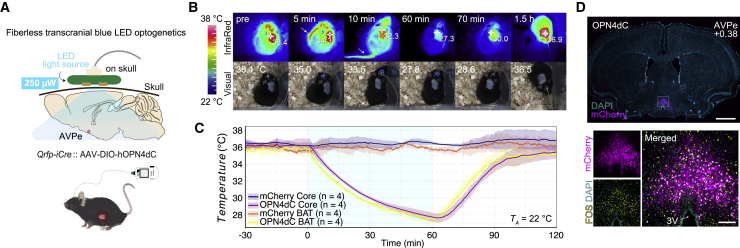
Transcranial manipulation of Q neurons (A) Fiberless transcranial blue LED stimulation in Q-OPN4dC mice. The LED source was attached on the mouse skull. The light intensity was set at 250 μW. (B) Infrared and visual imaging of a Q-OPN4dC mouse showing QIH. LED light was given transcranially to Q neurons expressing OPN4dC (250 μW, 1 h). The arrows in the 5 and 10 min figures indicate the vasodilation at the mouse tail. (C) Changes in *T*_*BAT*_ and *T*_*B*_ during the LED light exposure (250 μW, 1 h) in Q-OPN4dC mice and Q-mCherry control mice (n = 4 mice/group). (D) Representative tissue images showing the expressions of OPN4dC-mCherry (magenta) and c-Fos (yellow) as well as nuclei stained with DAPI (cyan) in the AVPe 60 min after the initiation of blue LED illumination (250 μW). Scale bars, 1 mm (top) and 100 μm (bottom). The lines and shading in (C) denote the means and SD, respectively. See also Video S2.

The high photosensitivity of OPN4dC inspired us to examine whether transcranial light delivery using an LED device could activate OPN4dC expressed in the AVPe to non-invasively induce QIH. A small blue LED light source was positioned directly above the surface of the skull of Q-OPN4dC mice (Figure 4A). Illuminating blue light (470 nm, 1 mA current, 250 μW power) caused a decrease in both *T*_*BAT*_ and *T*_*B*_ in Q-OPN4dC mice, although the efficiency was slightly weaker than that of direct stimulation of Q neurons via an optic cannula (Figures 4B and 4C; Video S2). The control group (Q-mCherry) exhibited no changes in body temperature, indicating that LED illumination itself had no effect on the body temperature under this condition. We confirmed c-Fos expression in OPN4dC-positive Q neurons following transcranial LED exposure (Figure 4D). This light power was low enough to not cause significant heat production in the LED device under this condition (Figure 4B). Importantly, fiberless stimulation of OPN4dC can be applied to other types of neurons. Figure S3D demonstrates that transcranial stimulation can excite hippocampal neurons, although it remains to be elucidated whether this stimulation indeed modulates physiology or behavior in animals.

Video S2. Non-invasive stimulation of hOPN4dC by a blue LED, related to Figure 4 Representative thermal and visual imaging of a *Qrfp-iCre* mouse expressing hOPN4dC-mCherry in the AVPe, during optogenetic stimulation via a blue LED attached on the skull (250 μW, 1 h from time 0). Core body temperature was simultaneously taken by telemetry system. Video plays at 60x speed. Images in Figure 4B are created from this video.

### OPN4dC optogenetics reveals that cardiovascular function is actively regulated by Q neurons

**Figure 5:**
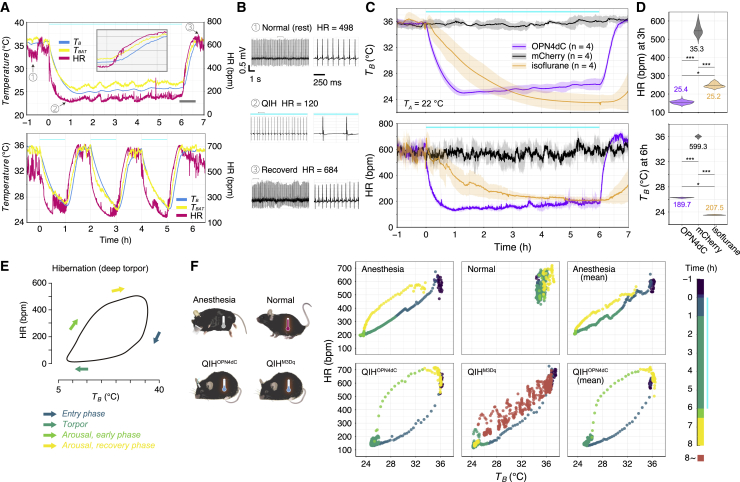
QIH^OPN4^ induces physiological changes similar to natural hibernation (A) Top, dynamics of HR, *T*_*B*_, and *T*_*BAT*_ during QIH^OPN4dC^ (n = 1). OPN4dC expressed in Q neurons was activated using a 473 nm laser (3 μW, 6 h). The initiation of light exposure was denoted as time 0. Inset represents a magnified view at the end of the light illumination, wherein the time range is indicated by a gray bar. Bottom, short-term stimulation (3 μW, 1 h) of OPN4dC with a good reproducibility in the effect on *T*_*B*_, *T*_*BAT*_, and HR. One h QIH was induced repeatedly every 2 h (n = 1). The HR and *T*_*B*_ were measured constantly via a telemetry system. (B) Electrocardiogram trace and HR from each condition, normal/pre-QIH (top), QIH (middle), and QIH mice during recovery phase (bottom). Electrocardiogram tracings for 10 and 1 s. (C) Comparison of *T*_*B*_ and HR between QIH^OPN4dC^ and isoflurane anesthesia. Q-mCherry mice were used as the controls (n = 4 mice/group). (D) Violin plots of HR (2.5–3.5 h after the QIH induction) when *T*_*B*_ values were approximately at the same level in the two conditions and of *T*_*B*_ (5–6 h after the QIH induction) when HR values were at approximately the same level in the two conditions. (E) Pattern diagram of scatterplots for *T*_*B*_ and HR of a Syrian hamster during hibernation (modified from a previous report^22^) typically forms an open loop. (F) The relationship of *T*_*B*_ and HR in several kinds of hypothermic state in mice. In QIH^OPN4dC^, Q neurons expressing OPN4dC were stimulated by blue light (3 μW, 6 h). In normal condition, Q neurons expressing mCherry were stimulated by the same condition as QIH^OPN4dC^. In QIH^M3Dq^, Q neurons expressing hM3Dq were excited by CNO intraperitoneal administration (0.1 mg/kg). In anesthesia, the same mice that were used in QIH^OPN4dC^, and normal conditions were exposed to 1% isoflurane for 6 h without photostimulation. The start time of light exposure, CNO intraperitoneal administration, and isoflurane inhalation were set to time 0. HR for each condition is plotted as a function of the concurrent *T*_*B*_. The plots are presented by each color for 1 h before the stimulus onset, for 6 h of stimulation, and for 2 h of recovery. In QIH^M3Dq^, extra plots of the time to complete recovery are also presented (orange, 8–24 h), as no clear recovery is observed even 6 h after the initiation of stimulation. The lines and shading in (C) denote the mean and SD of each group, respectively. All p values (∗p < 0.01, ∗∗p < 0.001, ∗∗∗p < 0.0001) are from one-way ANOVA with Tukey’s multiple comparisons test.

Hibernators arise every several days, i.e., interbout arousal (IBA), during which the body temperature rapidly returns to approximately 37°C and is maintained within the normal range for several hours.^41^ They return to deep hibernation and sustain low body temperatures until subsequent IBA. In terms of drastic physiological transitions from the normal to torpid state (and vice versa), we need to know how the central and peripheral nervous systems behave in each step (induction/entry, torpor, and recovery/IBA).^42^ Using QIH^OPN4dC^, we mimicked these states and interstate transitions in mice. We examined the changes in vital signs, including *T*_*B*_, *T*_*BAT*_, electrocardiogram (ECG), and heart rate (HR), with high temporal resolution. QIH^OPN4dC^ mice showed a rapid decrease in HR immediately after the start of light illumination (3 μW, 6 h, same as Figure 1C), followed by a reduction in *T*_*B*_ and *T*_*BAT*_ (Figures 5A and 5B). Likewise, during the recovery phase, HR increased first, and then *T*_*B*_ and *T*_*BAT*_ slowly returned to basal levels. This result suggests that changes in cardiovascular function in the QIH are not secondary to changes in body temperature. Repeated short-term stimulation (3 μW, 1 h laser exposure with an interval of 1 h, three times) resulted in similar fluctuations in HR with high reproducibility (Figure 5A). We used general anesthesia (isoflurane inhalation) as a control condition with hypothermia for comparison. During QIH^OPN4dC^, HR was decreased more rapidly and deeply compared to during anesthesia (Figure 5C). Even when *T*_*B*_ was almost identical in the two groups (at 2.5–3.5 h after beginning of stimulation), HR in QIH^OPN4dC^ was lower than that in anesthesia (Figure 5D). Similarly, when *T*_*B*_ in QIH^OPN4dC^ was higher than that in anesthesia (5–6 h after the beginning of stimulation), their HRs were almost the same in both conditions (Figure 5D). These results demonstrate that changes in HR are directly regulated by Q neurons and are not secondary to decreased body temperature. The HR right after the recovery from QIH^OPN4dC^ was higher than that in the basal condition (Figures 5B and 5C). HR overshoot is also observed in the natural hibernation of hibernators, such as the Syrian hamster.^22^ We then compared the kinetics of *T*_*B*_ and HR between QIH^OPN4dC^, QIH^M3Dq^, and isoflurane-induced anesthesia (Figure 5F). In the hibernation of natural hibernators, upon entering torpor, HR initially decreased rapidly, followed by a more gradual and delayed decline in *T*_*B*_, followed by a more gradual decrease in HR as *T*_*B*_ decreases rapidly^22^ (Figure 5E). Therefore, the scatterplot of these parameters generally takes the form of an open loop^22^ (Figure 5E). QIH^M3Dq^ also induced a rapid decrease in *T*_*B*_ and HR; however, these two factors were highly correlated (Figure 5F). In anesthetized mice, the HR-*T*_*B*_ loop was more flattened, indicating that HR variability closely correlates with *T*_*B*_. In QIH^OPN4dC^, cardiac function was suppressed in advance of the *T*_*B*_ decrease, and the offset timing was completely controllable (Figure 5F). In addition, the physiological kinetics in QIH^OPN4dC^ summarized by the plot of *T*_*B*_ and HR were similar to those of hibernation (Figures 5E and 5F). Overall, these results demonstrated that QIH^OPN4dC^ is similar to natural hibernation in terms of body temperature and cardiovascular regulation.

## Discussion

The present study showed that OPN4dC is useful for the long-term manipulation of neurons. While the development of novel optogenetic tools has provided researchers with optimized methods for neural manipulation, almost all applications of these opsins have been applied for a short period (usually seconds to minutes), and few studies have examined their effects on long-term (sometimes lasting more than 1 day) physiological changes. DREADD is usually used for long-term manipulation rather than optogenetics, while the termination of DREADD-mediated manipulation is dependent on the clearance of CNO *in vivo*. Thus, the OPN4dC method has two advantages: a long-term effect, such as DREADD, and high temporal resolution. Another characteristic of OPN4dC optogenetics is that it induces Gq signaling. Optogenetics using long-wavelength vertebrate cone opsin with C-terminal domains of 5-HT_1A_ receptor^43^ and engineered mosquito OPN3^44^ reportedly activates Gi signaling, resulting in inhibitory action on neurons. For Gq signaling, Opto-α_1_AR, a chimeric protein of bovine rhodopsin and the α_1A_-adrenergic receptor, was developed.^45^ However, as vertebrate rhodopsin is a monostable opsin, bleaching during long-term application is a concern. Indeed, cortical astrocytes expressing Opto-α_1_AR required supplement with 9*-cis* retinal to work properly.^46^ Therefore, we used human OPN4, a bistable opsin, to induce a hibernation-like state.

Ectopic expression of OPN4 has been used for trials of optogenetics-based therapy for retinal degeneration^24^^,^^47^ because of its high photosensitivity to visible blue light and sluggish phototransduction in cells.^48^^,^^49^ The full-length OPN4 has also been used in a few studies for manipulating Gq signaling in neurons^27^^,^^28^ and astrocytes.^50^^,^^51^ For instance, the excitation of orexin neurons using OPN4 with blue light (19 mW, 5 s) promoted wakefulness.^28^ However, the authors of that paper failed to manipulate neurons in the hippocampus using the same method,^52^ suggesting that there is something missing to make OPN4 work efficiently. We found that the truncation of C-terminal region of OPN4 significantly increased the activity of Gq signaling (Figure S2), presumably by decreasing internalization and degradation, and, consequently, the effect on body temperature was enhanced (Figure 1B). Another point that should be considered is that the commonly used light power (3–30 mW)^31^ is too high for OPN4 to function properly. Indeed, we observed that bright blue light (100 μW) was not as effective as weak light (10 μW; Figures 2B and 2C), although this mechanism needs to be elucidated in further studies using biochemical and electrophysiological assays. One possibility is the shortage of retinal in the brain tissue because we continuously exposed blue light to OPN4dC. However, endogenous OPN4 usually responds to strong light with high temporal resolution in ipRGCs of the retina, where the retinal can be efficiently reproduced. The slow kinetics of membrane potentials in OPN4dC-expressing neurons were similar to those of ipRGCs that express endogenous OPN4.^30^ The latency to evoke a spike after light stimulation was 6.09 ± 3.83 s, and the increase of firing rate continued after the end of stimulation, while the period seems to depend on the neuronal type (for >3 min in primary neuron culture, approximately 15 s in pyramidal neurons in the hippocampus and approximately 100 s in orexin-producing neurons^28^). Conversely, ChR2, a channel-type opsin, usually evokes spikes within several milliseconds, allowing precise control of the firing frequency by light pulse.^4^ We are able to select appropriate tools for the physiologies that we would like to examine according to the properties of opsins.

OPN5-expressing neurons in the POA were recently identified as inducing hypothermia by violet-light illumination in mice,^53^ and OPN5-positive neurons likely overlapped with Q neurons.^53^^,^^54^ Considering the expression of such endogenous photosensitive proteins, stimulating light with appropriate ranges of intensity and wavelength must be used. Under the conditions used in this study, we did not observe any effect of blue-light stimulation on *T*_*BAT*_ in the control mice (Figure 1C). For long-term manipulation, light-induced tissue injury should also be considered. Exposure to 3–30 mW light could induce glial activation, a marker of inflammation in the brain,^8^^,^^55^ and we observed the induction of Iba1 and GFAP expression with 10 mW light stimulation (2 Hz, 10 ms width, 24 h; Figure S5). The present study showed that the light power used in most of the experiments for evoking QIH was 3 μW (100 μW mm^−2^), but 0.1 μW (3.2 μW mm^−2^) blue laser was sufficiently strong to induce QIH (Figures 2B and 2C). The extremely low light intensity facilitates more localized light stimulation than has been achieved so far.

We also succeeded in stimulating them by transcranial, fiberless illumination of weak blue LED light (250 μW), even though Q neurons are in the deep brain. Several groups have developed methods for transcranial optogenetics.^56^^,^^57^^,^^58^ For example, highly sensitive ChR such as ChRmine^8^ and SOUL^55^ have been applied for transcranial optogenetics. Here, we have added OPN4dC optogenetics to the list of non-invasive methods. Considering that LED are quite easy to process in terms of their shape and size, we also emphasize the usefulness of OPN4dC for photostimulation by an LED in peripheral tissues besides the CNS. Indeed, channels and GPCR optogenetics have been used in autonomic neurons of the heart.^59^

In summary, the proposed method has several advantages. (1) Its effect was long lasting and stable for at least 24 h with good reproducibility. (2) OPN4dC is sensitive and enables non-invasive manipulation. (3) OPN4dC is a metabotropic receptor that mimics the slow kinetics of G protein signaling in neurons (and likely other types of cells) and provides researchers with a proper option for neural manipulation involved in particular physiological functions. As an example of OPN4 optogenetic application, we demonstrated the fluctuation of cardiovascular function during all steps of QIH in mice. OPN4-based techniques may be an excellent tool to address the mechanisms of hibernation and other physiologies in the future.

### Limitations of the study

This study showed the sensitivity of the OPN4dC was sufficiently high for transcranial manipulation. However, because of concerns regarding heat production, we did not manipulate Q neurons by OPN4dC using blue LED over several hours. Further studies are needed to improve the sensitivity and/or expression levels of OPN4dC to establish long-term transcranial manipulation. The possibility that OPN4dC may be activated by room light or other light sources should also be considered. For OPN4dC expressed in Q neurons in the AVPe, we observed no effect on body temperature under room light (270–280 Lux white LED) when the mouse skull was completely covered with black skin. However, further characterization of OPN4dC as well as preparing a proper experimental control (e.g. mCherry-expressing control) will be necessary, especially when this method is applied to neurons in more superficial regions of the brain.

## STAR★Methods

### Key resources table

**Table:** 

REAGENT or RESOURCE	SOURCE	IDENTIFIER
**Antibodies**		

Rabbit polyclonal anti-cFos	EnCor Biotechnology	Cat# RPCA-*c*-FOS; RRID: AB_2572236
Goat anti-mCherry	SICGEN	Cat# AB0040-200; RRID:AB_2333093
Rat anti-GFP	Nacalai Tesque	Cat# 04404-84; RRID: AB_10013361
Goat anti-Iba1	WAKO	Cat# 011-27991
Rabbit anti-GFAP	ATLAS ANTIBODIES	Cat# HPA056030; RRID: AB_2683015

**Bacterial and virus strains**		

AAV10-EF1α-DIO-hOPN4-mCherry	This paper	N/A
AAV10-EF1a-DIO-hOPN4dC-mCherry	This paper	N/A
AAV10-EF1a-DIO-hOPN4-9A-mCherry	This paper	N/A
AAV10-EF1a-DIO-hOPN4-9A-dC-mCherry	This paper	N/A
AAV10-EF1α-DIO-mCherry	Takahashi et al. (2020)	N/A
AAV10-EF1α-DIO-hM3Dq-mCherry	Takahashi et al. (2020)	N/A
AAV10-EF1α-DIO-ChR2(H134R)-EYFP	Hasegawa et al. (2017)	N/A
AAV10-EF1α-DIO-SSFO-EYFP	Takahashi et al. (2020)	N/A
AAV10-hSyn-iCre	This paper	N/A
AAV10-hSyn-DIO-GCaMP6s	This paper	N/A

**Chemicals, peptides, and recombinant proteins**		

Isoflurane	Pfizer	Cat# 4987114133403
Clozapine N-oxide (CNO)	Abcam	Cat# ab141704
D-MEM(High Glucose) with L-Glutamine, Phenol Red and Sodium Pyruvate	Fujifilm Wako Pure Chemical Corp.	Cat# 041-30081
D-Luciferin Potassium Salt	Fujifilm Wako Pure Chemical Corp.	Cat# 126-05116
Penicillin-Streptomycin Solution (×100)	Fujifilm Wako Pure Chemical Corp.	Cat# 168-23191
PEI MAXTM - Transfection Grade Linear Polyethylenimine Hydrochloride (MW 40,000)	Polysciences	Cat# 24765-100
FuGENE® HD Transfection Reagent	Promega Corporation	Cat# E2311
Neuron Culture Medium	Fujifilm Wako Pure Chemical Corp.	Cat# 148-09671
Neuron Dissociation Solutions	Fujifilm Wako Pure Chemical Corp.	Cat# 291-78001
Cerebral Cortex, from Rat(embryonic day 17)	Fujifilm Wako Pure Chemical Corp.	Cat# 033-24871
Dulbecco’s Modified Eagle’s Medium - low glucose paraformaldehyde	Merck KGaA	Cat# D2902
Opti-MEM™ I Reduced Serum Medium	Thermo fisher scientific	Cat# 31985062

**Critical commercial assays**		

In-Fusion HD Cloning Kit	Clontech Laboratories, Inc	Cat# Z9648N
Nano-Glo® Endurazine Live Cell Substrates	Promega Corporation	Cat# N2571
PlasMem Bright Green	DOJINDO	Cat# P504
Fluo 4-AM	DOJINDO	Cat# F311
LightCycler® 480 Probe Master	Roche Diagnostics	Cat# 50-720-3179
Universal ProbeLibrary, Probe#77	Roche Diagnostics	Cat# 04-689-003-001
NucleoBond Xtra Midi EF	MACHEREY-NAGEL	Cat# 740420

**Experimental models: Cell lines**		

HEK293T	RIKEN BioResource Research Center	RCB2202
NIH/3T3	Cell Resource Center for Biomedical research/Cell Bank, Tohoku University	ID: TKG 0299
DH5a	TOYOBO	Cat# DNA-903F

**Experimental models: Organisms/strains**		

Mouse: C57BL/6J	Charles River Laboratories Japan	N/A
Mouse: Qrfp-iCre (B6J.B6N-Qrfp < tm2.1(icre)Stak)	Takahashi et al. (2020)	N/A
Mouse: Calb1-Cre (B6.129S-Calb1tm2.1(cre)Hze/J)	Jackson Laboratory	Cat# 028532
Mouse: EGFP-L10a (B6.129S4-Gt(ROSA)26Sortm1(CAG-EGFP/Rpl10a,-birA)Wtp/J)	Jackson Laboratory	Cat# 022367

**Oligonucleotides**		

Synthesized oligo nucleotides with mutations from serine/threonine to alanine (SA/TA) to create hOPN4-9A and hOPN4-9A-dC. 5′- AGTCGCCCCTACCCCGCCTACC GCGCCGCCCACCGCGCCGCCCTG GCCGCCCACGCCGCCAACCTCAG CTGGATCTCC-3′	This paper	N/A
Forward primer for titration of AAV vectors 5′- actgtgtttgctgacgcaac	This paper	N/A
Reverse primer for titration of AAV vectors 5′- agcgaaagtcccggaaag	This paper	N/A

**Recombinant DNA**		

Plasmid: pAAV-EF1a-DIO-hM3Dq-mCherry	Roth lab DREADDs (unpublished)	Addgene #50460
Plasmid: pAAV-GFAP104-melanopsin-mCherry	Mederos et al. (2020)	Addgene #122630
Plasmid: pAAV-EF1a-DIO-hOPN4-mCherry	This paper	N/A
Plasmid: pAAV-EF1a-DIO-hOPN4dC-mCherry	This paper	N/A
Plasmid: pAAV-EF1a-DIO-hOPN4-9A-mCherry	This paper	N/A
Plasmid: pAAV-EF1a-DIO-hOPN4-9A-dC-mCherry	This paper	N/A
Plasmid: pEF1α-EF1a-hOPN4-mCherry	This paper	N/A
Plasmid: pEF1α-EF1a-hOPN4-mCherry	This paper	N/A
Plasmid: pEF1α-mCherry-N1	Takara Bio Inc.	Cat# 631969
Plasmid: pcDNA3-Nano-lantern(Ca2+)_600	Saito et al. (2012)	Addgene #51982
Plasmid: pHelper Vector	Takara Bio Inc.	AAVpro® Helper Free System Cat#6230
Plasmid: pGL4.3-NFAT-RE-luc2	Promega Corporation	Cat# E8481

**Software and algorithms**		

PONEMAH Physiology Platform v.6.30	Data Sciences International.	https://www.datasci.com/
InfReC Analyzer NS9500 Professional	Nippon Avionics	https://www.avio.co.jp/
GraphPad Prism 9	GraphPad	http://Graphpad.com/
MATLAB	Mathworks	http://Mathworks.com/
Leica Application Suite X Life Science	Leica	https://www.leica-microsystems.com/
IGOR Pro version 6.0	WaveMetrics	https://www.wavemetrics.com/
Fiji/ImageJ	Schindelin et al. (2012)	https://fiji.sc/
R	The R Foundation	https://www.r-project.org/

**Other**		

Thermostatic chamber	SHINFACTORY	HC-100
optic cannula	KYOCERA Corporation	F0617S02A4PW060

### Resource availability

#### Lead contact

Further information and requests for resources and reagents should be directed to and will be fulfilled by the lead contact, Arisa Hirano (hirano.arisa.gt@u.tsukuba.ac.jp).

#### Materials availability

Plasmids generated in this study will be shared by the lead contact upon request.

### Experimental model and subject details

#### Animals

All animal experiments were performed at the International Institute of Integrative Sleep Medicine, University of Tsukuba, and at the Okinawa Institute of Science and Technology Graduate University in accordance with the guidelines for animal experiments and were approved by the animal experiments committees of the institutes. Animals accessed food and water *ad libitum* and were maintained at an ambient temperature of 22°C and a relative humidity of 50% with a 12-h light/12-h dark cycle. *Qrfp-iCre* knock-in mice (B6J.B6N-Qrfp < tm2.1(icre)Stak)^18^ and *Calb1-Cre* knock-in mice (B6.129S-Calb1tm2.1(cre)Hze/J, Jackson Laboratories stock #028532) have been previously described.^38^ The genetic background of *Qrfp-iCre* and *Calb1-Cre* mice was C57BL/6J. Male heterozygous *Qrfp-iCre* or *Calb1-Cre* mice were used for all experiments, unless otherwise indicated. All behavioral tests were performed at an ambient temperature of 22°C in a thermostatic chamber.

#### Cell lines

HEK293T cells and NIH/3T3 cells were obtained from RIKEN BioResource Research Center (Cell ID: RCB2202) and Cell Resource Center for Biomedical research/Cell Bank, Tohoku University (Cell ID: TKG 0299), respectively. Cells were cultured in Dulbecco’s Modified Eagle’s Medium (DMEM) (041–30081, FUJIFILM-Wako Pure Chemical Corp.) supplemented with 10% fetal bovine serum (FBS) (Sigma-aldrich) and penicillin-streptomycin solution (168–23191, FUJIFILM-Wako Pure Chemical Corp.) at 37°C, 5% CO2 in a humidified atmosphere. Primary cultures of neurons were prepared from the cerebral cortex of rat embryos (033–24871, FUJIFILM-Wako Pure Chemical Corp.) using a neuron dissociation solution (291–78001, FUJIFILM-Wako Pure Chemical Corp.). Neurons were cultured in the neuron culture medium (148–09671, FUJIFILM-Wako Pure Chemical Corp) at 37°C, 5% CO2 in a humidified atmosphere, and half the volume of the culture medium was replaced with fresh medium every 3 days. To prevent the growth of astrocytes, 5-mM AraC was added to the medium if necessary. For preparation of plasmids, *E. coli* DH5a obtained from TOYOBO (DNA-903F) was cultured in Lysogeny Broth medium supplemented with antibiotics at 30°C or 37°C. Plasmids were purified by using NucleoBond Xtra Midi EF (Cat# 740420, MACHEREY-NAGEL).

### Method details

#### Plasmid construction

To generate pAAV-EF1a-DIO-hOPN4-mCherry, pAAV-EF1a-DIO-hM3Dq-mCherry (addgene#50460) was modified. The coding sequences of hM3Dq was replaced by the sequence coding human OPN4 isoform1 (Genbank: NM_033282) in pAAV-GFAP104-melanopsin-mCherry (addgene#122630).^50^ Amino acid from 385 to 478 and from 400 to 478 was deleted for hOPN4-dC and hOPN4-9A-dC, respectively. For hOPN4-9A and hOPN4-9A-dC, synthesized oligo nucleotides with mutations from serine/threonine to alanine (SA/TA) were inserted into pAAV-EF1a-DIO-hOPN4-mCherry by In-Fusion HD Cloning Kit (Z9648N, Clontech Laboratories Inc.) at the position of amino acid 384, 387, 388, 391, 392, 394, 395, 397 and 398. pEF1α-hOPN4-mCherry and pEF1α-hOPN4dC-mCherry was generated by insertion of the coding sequences of hOPN4-mCherry and hOPN4dC-mCherry into pEF1α-mCherry-N1 (Clontech Laboratories Inc) by In-Fusion HD Cloning Kit (Z9648N, Clontech Laboratories Inc). pGL4.30-NFAT-Luc vector was purchased ([luc2P/NFAT-RE/Hygro], E8481, Promega Corp.).

#### Viral vector reparation

Virus vectors were generated by AAVpro Helper Free System (Takara Bio Inc.). 293T cells were transfected with pHelper plasmid, rh10 and pAAV plasmids with polyethylenimine (24,765-100, Polysciences,Inc.). Cells were harvested and resuspended in artificial cerebrospinal fluid (aCSF; 124 mM NaCl, 3 mM KCl, 26 mM NaHCO_3_, 2 mM CaCl_2_/2H_2_O, 1 mM MgSO_4_/7H_2_O, 1.25 mM KH_2_PO_4_, 10 mM D-glucose). After repeated freeze-thaw four times, cell suspension was centrifuged at 14 krpm at 4°C for 15 min. Supernatant was collected as virus solutions. The final purified virus vectors were stored at −80°C. Titer of recombinant AAV vectors was quantified by Taqman real-time PCR (50-720-3179, Roche Diagnostics). Primers and probe are listed in the key resources table. pAAV-EF1a-DIO-hM3Dq-mCherry, pAAV-EF1a-DIO-ChR2(H134R)-EYFP, pAAV-EF1a-DIO-SSFO-EYFP and pAAV-EF1a-DIO-mCherry were described previously.^18^^,^^61^

AAV10-EF1α-DIO-hOPN4-mCherry-WPRE (AAV-DIO-OPN4-mCherry), 1.1×10^13^ genome copies (gc)/mL; AAV10-EF1α-DIO-hOPN4-dC-mCherry-WPRE (AAV-DIO-OPN4-dC-mCherry), 1.0×10^13^ gc/ml for behavioral analysis and 4.0×10^12^ gc/ml for primary neuron cultures; AAV10-EF1α-DIO-hOPN4-9A-mCherry-WPRE (AAV-DIO-OPN4-9A-mCherry), 9.5×10^12^ gc/ml; AAV10-EF1α-DIO-hOPN4-9A-dC-mCherry-WPRE (AAV-DIO-OPN4-9A-dC-mCherry), 1.1×10^13^ gc/ml; AAV10-EF1α-DIO-ChR2(H134R)-EYFP-WPRE (AAV-DIO-ChR2-EYFP), 1.11×10^12^ gc/ml; AAV10-EF1α-DIO-SSFO-EYFP-WPRE (AAV-DIO-SSFO-EYFP), 1.35×10^12^ gc/ml; AAV10-EF1α-DIO-hM3Dq-mCherry (AAV-DIO-M3Dq-mCherry), 1.64 × 10^12^ gc/ml; AAV10-EF1α-DIO-mCherry (AAV-DIO-mCherry), 1.44 × 10^12^; AAV10-hSyn-iCre-WPRE, 1.0×10^13^ gc/ml; AAV10-hSyn-DIO-GCaMP6s, 2.62 × 10^12^ gc/ml.

#### Stereotaxic surgery

*Qrfp-iCre* heterozygous mice (12–20 weeks old) were anesthetized with ventilated 1–2% isoflurane (Pfizer, cat# 4987114133,403) and affixed to a stereotaxic frame (David Kopf Instruments) while being kept warm by a heat pad. *Qrfp-iCre* mice were injected with 0.15 μL AAV bilaterally into the AVPe (coordinates relative to the bregma: +0.38 mm AP, ±0.20 mm ML, −5.10 mm DV) at a controlled rate (0.1 μL/min) using a needle syringe (Neuros Syringe 1701 RN, 33 gauge, HAMILTON) and a stereotaxic injector (Legato 130, KD Scientific). The needle’s position was maintained for 5 min after the injection. All AAV-injected mice were given a recovery period of at least three weeks before subsequent experiments.

To validate the effects of hOPN4s (Figures 1A–1C), the mice were injected with AAVs (AAV-OPN4, AAV-OPN4dC, AAV-OPN4-9A, AAV-OPN4-9A-dC, or AAV-mCherry as a control) into the AVPe. To compare different types of opsins (Figure S4), mice were injected with each AAV (AAV-OPN4dC, AAV-ChR2, AAV-SSFO, or AAV-mCherry).

For other optogenetic studies, mice were injected with each AAV (AAV-OPN4dC or AAV-mCherry). For chemogenetic experiments (Figures 3B and 5F), mice were injected with AAV-M3Dq into the AVPe.

For tetrode recording, *Calb1-Cre* mice were injected with 0.5 μL AAV-OPN4dC unilaterally into the hippocampal CA1 (−2.0 mm AP, +1.5 mm ML, −1.5 mm DV) and implanted with the microdrive. The tetrodes (14-nm diameter, nichrome wire, gold plated to 200–300 kΩ) in the microdrive were independently adjustable and arranged to surround the optic fiber (200 μm diameter, Thorlabs, FT200EMT) located in the center. The tip of the optic fiber was placed 2.0 mm posterior/1.5 mm lateral from the bregma and at the surface of the brain. One of the stainless steel screws attached to the skull was used as the electrical ground for recording. Postoperatively, animals were carefully monitored and i.p. injected with 0.5 mL of 10% sucrose solution (in saline) when a loss of body weight (>10%) was observed. In to 2–3 weeks postoperatively, the depth of the tetrodes was slowly and manually adjusted until sharp wave ripples and associated spikes could be stably observed. All adjustments were made in a familiar small plastic bucket (20-cm diameter) located in the recording room.

#### Behavioral tests

All behavioral experiments were performed in a temperature-controlled chamber (HC-100, SHINFACTORY) at stable ambient temperature set at 22°C, under a 12:12 h light-dark cycle (light ON from 8 a.m. to 8 p.m., 270–280 Lux white LED light). The mice were housed in clear synthetic resin cages (25 × 20 × 19 cm) with aspen chips. The mice were allowed *ad libitum* access to food and water. Mouse body temperature was monitored using an implantable telemetry system (DSI) and/or an infrared camera, as described below. As needed, mouse movements were tracked using a video camera (FDR-AX60, SONY) installed above the cages.

#### Infrared thermography

Thermographic analyses were performed as previously described.^18^ Briefly, hair around the interscapular region of the subjects was shaved to measure the *T*_*BAT*_. Infrared thermographic images were obtained every 2 s (0.5 Hz) using an infrared camera (InfReC R500EX or R550S, Nippon Avionics) positioned 1 m above the bottom of the cage. The obtained images were analyzed using InfReC software (NS9500Pro, Nippon Avionics). The highest values every 10 s were used for data representation.

#### Telemetric vital signs monitoring

Core body temperature (*T*_*B*_) and HR were assessed using a telemetry system (PhysioTel and PONEMAH, DSI), as previously described.^18^ In brief, to record the T_B_ and ECG continuously, a telemetry temperature transmitter (TA11TA-F10 or TA11ETA-F10, DSI) was implanted in the abdominal cavity of mice under isoflurane anesthesia for at least seven days before the recording. Locomotor activity (*Act*) was monitored concurrently with *the T*_*B*_ (Figure 3B). For ECG measurements, two wires from the transmitter (TA11ETA-F10) were placed on the surface of the thoracic cavity. The ECG was analyzed with the PONEMAH Physiology Platform v.6.30 software (DSI) to calculate HR. Telemetry data were collected every 10 s (0.1 Hz) and used for data representation.

##### Correlation between T_B_ and HR with anesthesia

*The T*_*B*_ and HR were simultaneously recorded (Figure 4C) using the method described above.

Animals in the “QIH^OPN4^” and “Normal” groups were *Qrfp-iCre* mice that received AAV-hOPN4dC and AAV-mCherry into the AVPe, respectively. For anesthesia, the mice were administered 1% isoflurane for 6 h by using the inhalation anesthesia machine (MK-AT210, MUROMACHI KIKAI) at an ambient temperature of 22°C. After exposure to anesthesia for 6 h, the mice were immediately returned to the chamber in which they had been kept during the normothermic phase. Two mice were used for each group (“QIH^OPN4^” and “Normal”). The order of the experiments was randomized and unbiased.

##### T_B_-HR loop observation

The data used for the “QIH^OPN4^,” “Normal,” and “Anesthesia” were the same as the data presented in Figure 4C. Animals in the “QIH^M3Dq^” were *Qrfp-iCre* mice that received AAV-M3Dq into the AVPe. The time of each phase (Figure 4F) was designated as follows: -1–0 h: normothermic phase, 0–1 h: entry phase, 1–6 h: torpor phase, 6–7 h: arousal entry phase, and 7–8 h: arousal recovery phase. The “0 h” timepoint denoted the onset time of the photostimulation, isoflurane inhalation and CNO (ab141704, Abcam) intraperitoneal administration (ZT4). For QIH^M3Dq^, recording was continued until the mice had fully recovered (8 h-).

#### Optogenetic manipulation

AAV-injected mice were implanted with an optic cannula (200-μm diameter, NA:0.50, 6.0 mm long, KYOCERA) unilaterally above the AVPe (+0.38 mm AP, 0.00 mm ML, −4.80 mm DV). To stimulate the axon terminal (Figures 2G and 2H), the optic cannula was unilaterally implanted above the DMH (−1.58 mm AP, +0.25 mm ML, −4.75 mm DV). The fiberoptic cannulas implanted in mice were connected to a fiberoptic patch cable (200-μm diameter, NA:0.22, 1.0-m long, Doric Lenses) using ceramic sleeves (Thorlab). We used DPSS lasers (473 nm blue, BL473T8-100FC, Shanghai Laser) for the optogenetic manipulation. The intensity of the output laser at the optical fiber tip was measured using a laser checker (Coherent) and was adjusted to 3 μW (100 μW mm^−2^), unless otherwise indicated in the figure legend. Irradiation (μW mm^−2^) was calculated based on the light power at the fiber tip using “predicted irradiance values” (https://web.stanford.edu/group/dlab/cgi-bin/graph/chart.php). Laser stimulation was applied continuously for 6 h, controlled by a TTL pulse generator (Amuza), except when noted. To verify the light intensity at which hOPN4dC responded sufficiently to induce deep hypothermia, the laser power was set to 0.1, 1, 3, or 10 μW (Figure 2B). To stimulate ChR2 and SSFO (Figure S4), a laser was applied at 10 Hz for 10 ms-width for 6 h and at 1 Hz for 1 s width every 30 min for 6 h. To verify the spectral sensitivity of OPN4dC *in vivo* (Figure 2D), the laser powers (473, 532, 589, and 632 nm, Shanghai Laser) were set to 10 μW. To test the responses of OPN4dC to various stimulus frequencies (Figure 2E), 473 nm laser power was set to 10 μW and photostimulation was applied at each frequency (0.1, 1, 2, and 10 Hz) for 10-ms-width for 6 h.

Mice were habituated to the cages overnight prior to the optogenetic experiments. Laser stimulation was initiated at ZT4 (0 p.m.), except for Figure 3, in which the laser stimulation started at ZT6 (2 p.m.) and was applied for 24 h. After the experiment, the positions of the implanted cannula and gene expression were confirmed using immunohistochemistry. The data were included only if proteins were expressed in the AVPe by AAV infection and optic cannulas were implanted in the precise position.

#### Transcranial fiberless optogenetics

A laboratory-made small LED light source device (8-mm diameter, 4-mm height, 0.12 g weight) was attached directly to the surface of the skull (around the bregma) of Q-OPN4dC and Q-mCherry control mice using dental cement and covered with the scalp. The device was equipped with two commercially available blue LED chips (center wavelength: 470 nm, ISO 0603 size). The LEDs were molded with transparent epoxy resin. The LEDs on the device were driven via current-controlled operation by a lab-made battery-powered LED driver equipped with a microcontroller and a Bluetooth chip. It could control the period and intensity of LED light using lab-made control software on a PC. The current was set to 1.0 mA and the output power of the LED was measured as 250 μW. After two days of recovery from the surgery, the LED device on the mouse head was connected to the LED driver with a patch cable. The mice were allowed to move freely in and habituate to the experimental cages for 1 h before light exposure.

#### Tetrode recording and analysis procedures

Two to three weeks postoperatively, recording sessions were conducted in a familiar bucket. All the data were acquired using a 32-channel Digital Lynx 4SX acquisition system (Neuralynx). The local field potential (LFP) was filtered at 2–9,000 Hz. Spike waveforms were filtered between 0.6 and 6 kHz and those above a peak threshold of 50 V were time-stamped and digitized at 32,556 Hz. The optical fiber in the microdrive was coupled to a 473 nm DPSS laser (Shanghai Laser & Optics Century, BL473T8-300FC). Ten minutes after the baseline recording, blue light (10 mW, 10-s duration, 10-min inter-stimulus interval, 10 trials) was delivered while recording the spikes and LFP.

The multi-unit activity was analyzed using scripts written in MATLAB (MathWorks). Firing rates during the 10 s pre- or post-light onsets were calculated for the OFF or ON epochs. For each electrode, the mean firing rates during the OFF epochs were used to normalize the firing rates of both the OFF and ON epochs for pairwise comparison across the electrodes.

#### Transfection

HEK293T cells were transfected with polyethylenimine (24,765-100, Polysciences,Inc., stock solution: 1 mg/mL polyethylenimine dissolved in water, pH 7.4). NIH3T3cells were transfected with FuGENE HD transfection reagent (E2311, Promega corp.). DNA and the transfection reagents were incubated in the opto-MEM (31,985,062, Thermo fisher scientific) at 23°C.

#### Membrane localization of hOPN4dC

HEK293T cells seeded on a cover glass were transfected with the expression construct, pEF1α-hOPN4-mCherry or pEF1α-hOPN4dC-mCherry. Twenty-four hours after transfection, the cellular membranes were stained with PlasMem Bright Green (P504, DOJINDO). Images were obtained using a laser confocal microscope (TCS SP8, Leica Biosystems).

#### Intracellular Ca^2+^ measurement in HEK293T cells

For fluorescence-based Ca^2+^ recording, HEK293T cells were transfected with the expression construct, pEF1α-hOPN4-mCherry, pEF1α-hOPN4dC-mCherry, or pEF1α-mCherry-N1. Twenty-four hours after the transfection, the medium was changed to the recording medium: DMEM (D2902, Merck KGaA) supplemented with 10% FBS, 3.5 mg/mL glucose, 25 U/mL penicillin, 25 μg/mL streptomycin, and 10 mM HEPES-NaOH, pH 7.0. On the second day after transfection, Fluo-4AM (F311, DOJINDO) was added to the cells 1 h before recording, following the manufacturer’s protocol. Fluorescence was detected using a microplate reader (Infinite 200 PRO, TECAN) at an interval of 2 s (Ex. 480 nm/Em 519 nm) at 37°C. Stimulating light for hOPN4s and excitation light for fluorescence were the same (480 nm, 500 ms, every 2 s), as described in a previous study.^29^^,^^62^

For bioluminescence-based Ca2+ recording, cells were transfected with the expression constructs pEF1a-hOPN4s-mCherry and pcDNA3-Nano-lantern(Ca2+)_600 (Addgene, #51982^63^). Twenty-four hours after transfection, the cell culture medium was replaced with recording medium. Twenty-four hours after the medium change, the substrate of luciferase (Endurazine, N2570, Promega) was added to the recording medium following the manufacturer’s protocol. Bioluminescence was measured using the GloMax Discover Microplate Reader (Promega corp.) at 37°C. Between the detection of bioluminescence, blue-light illumination (475 nm, 300 ms, every 6 s) was provided to the cells five times.

#### NFAT-luc assay in cultured cells

The pGL4.30-NFAT-Luc vector and hOPN4 expressing vectors were transfected into NIH3T3cells seeded in a glass-bottom black 24-well plate. Twenty-four hours after the transfection, the culture medium was replaced with the recording medium (DMEM (D2902, Merck KGaA) supplemented with 10% FBS, 3.5 mg/mL glucose, 25 U/mL penicillin, 25 μg/mL streptomycin, 0.2 mM luciferin and 10 mM HEPES-NaOH; pH 7.0.) and bioluminescence was recorded using Chronos HT (ATTO) in air at 37°C. Blue LED light (475 nm), green LED (520 nm), and violet LED (405 nm) were used for photostimulation. The expression levels of hOPN4-mCherry and hOPN4dC-mCherry were examined by measuring the fluorescence signals of mCherry using a plate reader (Promega corp.).

#### Immunohistochemistry

Tissue preparation, sectioning and staining for immunohistochemistry were performed as previously described.^18^ In brief, animals were deeply anesthetized with isoflurane and transcardially perfused with chilled 4% paraformaldehyde/PBS solution. For immunofluorescence, brains were dissected, post-fixed in 4% paraformaldehyde/PBS overnight at 4°C, incubated overnight in sucrose solution and embedded in Tissue-Tech O.C.T. compound (45,833, Sakura Finetech). Thirty μm-thick sections were obtained with a cryostat (CM1860, Leica) and subsequently processed for immunofluorescence. Primary antibodies used for the immunostaining were rabbit anti-cFos (1:2,000, AB2572236, EnCor Biotechnology), goat anti-mCherry (1:15,000, AB0040-200, SICGEN), rat anti-GFP (1:5,000, 04,404-84, Nacalai Tesque), goat anti-Iba1 (1:100, 011–27991, WAKO) and rabbit anti-GFAP (1:2000, HPA056030, ATLAS ANTIBODIES). Secondary antibodies were Alexa Fluor 488 donkey anti-rat, 488 donkey anti-rabbit, 594 donkey anti-rabbit and 594 donkey anti-goat (1:1,000, Invitrogen). For nuclei staining, sections were counterstained using 4′,6-diamidino-2-phenylindole (DAPI) (Cellstain- DAPI solution, D523, 1:5000, Dojindo) during the first washing step with PBS after the secondary antibody reaction. Brain sections were observed by using a laser confocal microscope (TCS SP8, Leica Biosystems).

#### Counting of c-Fos positive neurons

Images of the brains expressing c-Fos were obtained using a laser confocal microscope (TCS SP8, Leica Biosystems). mCherry- and/or c-Fos-expressing neurons were manually counted in each brain section (+0.38 mm AP from bregma) of mice (n = 3/group) using the LAS X software platform (Leica Biosystems). Figure 1B shows the ratio of mCherry^+^/c-Fos^+^ neurons to mCherry^+^ single positive neurons.

#### Validation of OPN4dc expression in Q neurons

The *Qrfp-iCre* mice were crossed with Rosa26-CAG-loxP-STOP-loxP-Rpl10a-EGFP (EGFP-L10a; B6.129S4-Gt(ROSA)26Sor^tm1(CAG−EGFP/Rpl10a,−birA)Wtp/J^; Jackson Laboratories stock #022367) reporter mice. The *Qrfp-iCre; EGFP-L10a* mice were injected with AAV-DIO-OPN4dC-mCherry into the AVPe. Three weeks after viral injection, the mice were sacrificed, and brain samples were subjected to immunohistochemistry. Fluorescent signals from confocal images were counted manually.

#### Histological confirmation of tissue injury

Optic fibers were bilaterally implanted into the lateral hypothalamic area (LHA) of WT C57B6/J mice without viral injection. Four weeks after, the mice were divided into four groups and 473 nm light stimulation were given unilaterally for 24 h: “OPN4” (continuous 10 μW light); “ChR2” (10 mW, 2 Hz, 10-ms-width); and “SSFO” (1 s or 3 s) (10 mW, 1s- or 3-s width, every 30 min) (n = 3/group). The mice were sacrificed 48 h after photostimulation. Coronal brain sections, including the LHA, were prepared and subjected to immunohistological analysis using anti-Iba1 and anti-GFAP antibodies. Iba1-and GFAP-positive cells in the region of interest (ROI) (1-mm square, around the optical fiber tip in the lateral hypothalamus) of the images were counted manually.

#### Electrophysiology of primary neurons

From third to fifth days of culture, primary neurons were infected with AAV-DIO-OPN4dC-mCherry and AAV10-hSyn-iCre-WPRE (MOI:2.0 × 10^5^ for each AAV). For electrophysiological recordings, neurons were cultured for over 20 days for maturation. Primary neurons in culture infected with AAV10-EF1a-DIO-hOPN4dC-mCherry and AAV10-hSyn-iCre were incubated in artificial cerebrospinal fluid (aCSF; 125 mM NaCl, 26 mM NaHCO_3_, 10 mM D(+)-glucose, 2.5 mM KCl, 2 mM CaCl_2_ and 1 mM MgSO_4_) bubbled with O_2_ (95%) and CO_2_ (5%) during the whole-cell patch clamp recording at 23°C. Responsiveness to blue light (150 μW/mm^2^ from the fiber chip, 20 s) was recorded in whole-cell current-clamp mode using K-gluconate-based internal solution (125 mM K-gluconate, 10 mM HEPES, 10 mM phosphocreatine, 0.05 mM tolbutamide, 4 mM NaCl, 4 mM ATP, 2 mM MgCl2, 0.4 mM GTP and 0.2 mM EGTA, pH 7.3, adjusted with KOH). The output signals were low-pass filtered at 3 kHz, digitized at 20 kHz using a Multi-Clamp 700 B amplifier and A/D hardware (National Instruments), and recorded using IGOR Pro software (version 6.0; WaveMetrics). The baselines of membrane potential without any photostimulation were obtained for at least 120-s recording. After photostimulation, the output signals were recorded for more 5 min to examine the effect of light. The firing rate and membrane potential were calculated every second from 20 s before photostimulation.

#### Ca2+ imaging in slice preparations

Two 9-week-old *Qrfp-iCre* mice injected with AAV-DIO-OPN4-dC-mCherry or AAV-DIO-GCaMP6s were used for Ca^2+^ imaging. Acute brain slices were prepared using a slightly modified N-methyl-*d*-glucamine (NMDG) protective recovery method.^64^ Briefly, mice were deeply anesthetized by isoflurane and then cardiovascular perfused with ice-cold NMDG-aCSF consisting of 93 mM NMDG, 93 mM HCl, 2.5 mM KCl, 1.2 mM NaH_2_PO_4_, 30 mM NaHCO_3_, 20 mM HEPES, 25 mM D-glucose, 5 mM Na-ascorbate, 2 mM thiourea, 3 mM Na-pyruvate, 10 mM MgSO_4_, and 0.5 mM CaCl_2_ (pH 7.4, adjusted with HCl, oxygenated with 95% O_2_/5% CO_2_). The brain was removed and cut at a thickness of 250 μm in ice-cold NMDG-aCSF using a vibratome (Leica VT1200S) under dim light. Slices containing the AVPe were incubated in the NMDG-aCSF at 32°C for 12 min and then in HEPES-aCSF consisting of 24 mM NaCl, 2.5 mM KCl, 1.2 mM NaH_2_PO_4_, 24 mM NaHCO_3_, 5 mM HEPES, 12.5 mM D-glucose, 2 mM CaCl_2_, and 1 mM MgSO_4_ (∼310 mOsm, oxygenated with 95% O_2_/5% CO_2_) at 23°C for >1 h. Incubation was performed in the dark. Slices were transferred into a chamber under a fluorescent upright microscope (Zeiss AxioExaminer D1, HXP120×, 40× water-immersion objective lens, and zoom 0.5) and submerged in the perfused HEPES-sCSF at 32–35°C. Fluorescence signals were acquired using an EM-CCD camera (Andor iXon Ultra 888), in which GCaMP6s and mCherry fluorescence were observed using a Zeiss filter set 38HE (excitation 470/40 nm, emission 525/50 nm) and a Zeiss filter set 43 (excitation 545/25 nm, emission 605/70 nm), respectively. hOPN4dC-expressing neurons were identified using mCherry expression. For optical stimulation, an optical fiber was placed just above the OPN4-expressing area and outside the field of view. Ca^2+^ (GCaMP6s) signals were imaged using Andor iQ software (1024 × 1024 pixels, 0.2 fps, and exposure time of 20 ms). During Ca^2+^ imaging, 3-s optical stimuli (473 nm laser at 75 μW) were repeated 10 times at 5-s intervals with a function generator (Master-9, AMPI). The timing of the optical stimuli was outside the Ca^2+^ imaging frame. The timing of image acquisition and optical stimulation was monitored using a Digidata data acquisition system (Molecular Devices) and was used in the analysis. The Ca^2+^ signal of each Q-neuron in each frame (Ft) was estimated based on the mean intensity across the pixels within each ROI after subtraction of the background signal using Fiji/ImageJ software.^65^ The following analyses were performed using R software. The relative fluorescence signal (ΔF/F)_t_ was calculated for each ROI as follows:

(ΔF/F)_t_=(F_t_−F_o_)/F_o_, where F_o_ is the mean value of F_t_ for 10 frames before the start of optical stimulation.

To compare changes in Ca^2+^ signals between neurons, the (ΔF/F)_t_ of each neuron was standardized using values from 10 frames before the onset of the optical stimulus. The standardized ΔF/F is shown as the ΔF/F_*Z* score in the figure.

### Quantification and statistical analysis

Statistical analyses were performed using the GraphPad Prism 9. Group comparisons were performed using one-way ANOVA followed by post-hoc tests (Tukey’s test or Wilcoxon matched-pairs signed rank test). Statistically significant differences were established at ∗p < 0.01, ∗∗p < 0.001 and ∗∗∗p < 0.0001. NS indicated no statistically significant difference. The sample size for each experiment is reported in the article or figures. No statistical methods were used to predetermine sample size. Investigators were not blinded to allocation during experiments and outcome assessment. The experiments were not randomized.

## Data Availability

• All data reported in this paper will be shared by the lead contact upon request. • This paper does not report original code. • Any additional information required to reanalyze the data reported in this paper is available from the lead contact upon request.

## Supplemental information

Document S1. Figures S1–S5

Document S2. Article plus supplemental information
