# Supplementary material for: Optogenetic induction of hibernation-like state with modified human Opsin4 in mice
Source: Cell Rep Methods. 2022 Nov 14;2(11):100336. doi: 10.1016/j.crmeth.2022.100336 (PMC9701604; doi:10.1016/j.crmeth.2022.100336)
Supplement: Document S1. Figures S1–S5 [file mmc1.pdf]

Cell Reports Methods, Volume *2*

Supplemental information

Optogenetic induction of hibernation-like state with modified human Opsin4 in mice

Tohru M. Takahashi, Arisa Hirano, Takeshi Kanda, Viviane M. Saito, Hiroto Ashitomi, Kazumasa Z. Tanaka, Yasufumi Yokoshiki, Kosaku Masuda, Masashi Yanagisawa, Kaspar E. Vogt, Takashi Tokuda, and Takeshi Sakurai

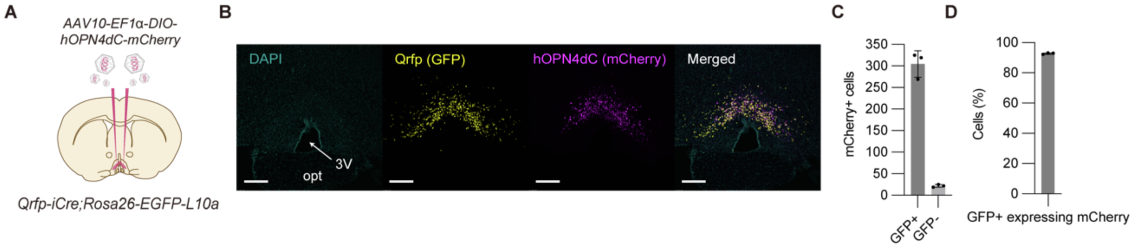

Figure S1. Validation of hOPN4dC expression in Q neurons, related to Figure 1.

(A and B) Immunohistochemistry experiments demonstrate co-localization of recombinase activity in *Qrfp-iCre* mice and Cre-dependent AAV-mediated hOPN4dC-mCherry expression. (A) AAV10-EF1α-DIO-hOPN4dC-mCherry was injected into the AVPe of double transgenic animals *Qrfp-iCre; Rosa26-LSL-GFPL10a*. (B) Representative immunohistochemistry image for mCherry (hOPN4dC-mCherry) and GFP in *Qrfp-iCre; Rosa26-LSL-GFPL10a* double transgenic mice. Scale bars: 200 µm.

(C and D) Bar graphs show cell number of mCherry-positive (mCherry+) cells that express GFP (C) and percentages of GFP-positive (GFP+) cells that express mCherry (D). GFP- means GFP-negative.

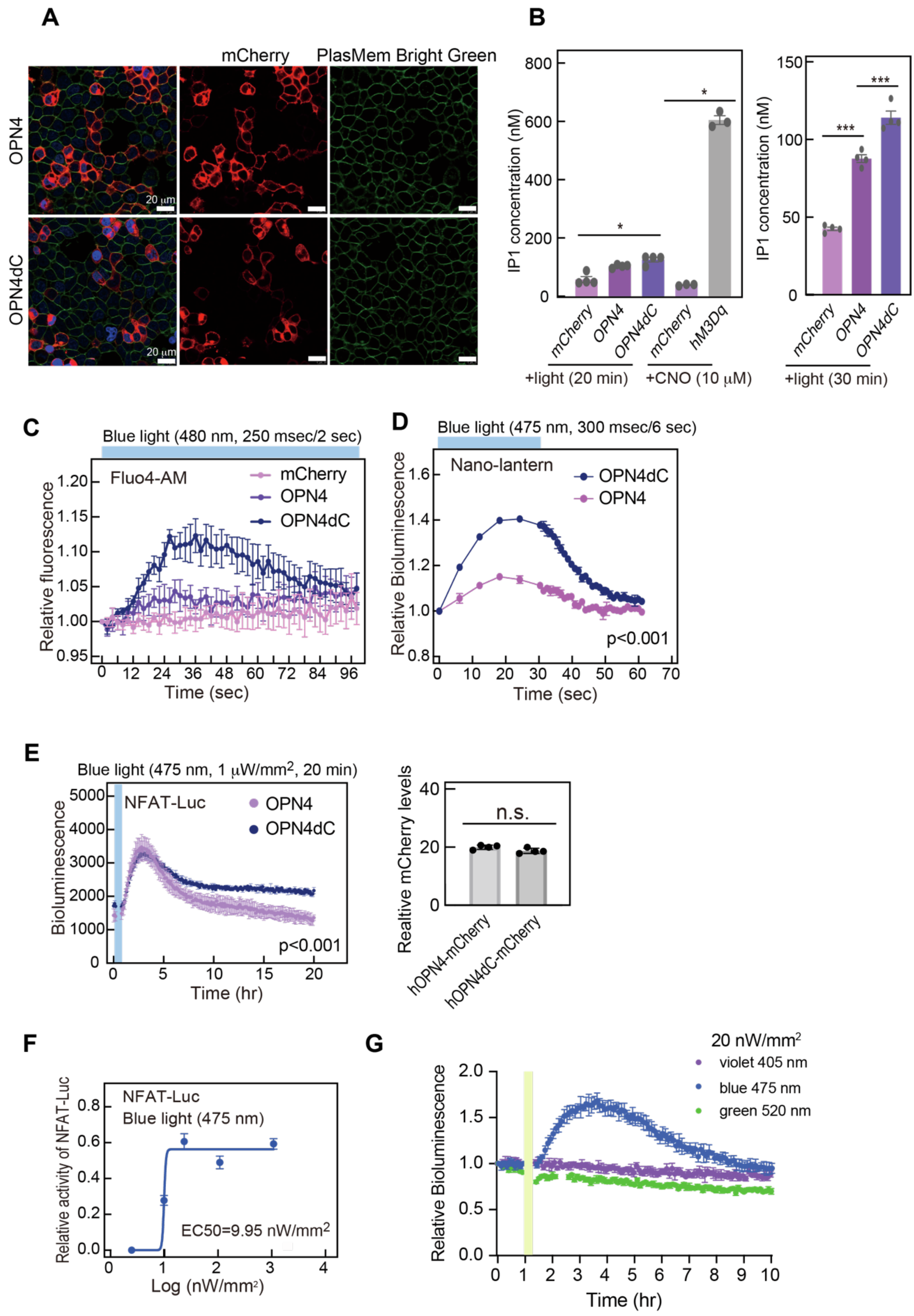
A
mCherry
PlasMem Bright Green
OPN4
OPN4dC
20 μm
B
IP1 concentration (nM)
mCherry
OPN4
OPN4dC
mCherry
hM3Dq
+light (20 min)
+CNO (10 μM)
+light (30 min)
C
Blue light (480 nm, 250 msec/2 sec)
Fluo4-AM
mCherry
OPN4
OPN4dC
Relative fluorescence
Time (sec)
D
Blue light (475 nm, 300 msec/6 sec)
Nano-lantern
OPN4dC
OPN4
Relative Bioluminescence
p<0.001
Time (sec)
E
Blue light (475 nm, 1 μW/mm2, 20 min)
NFAT-Luc
OPN4
OPN4dC
Bioluminescence
p<0.001
Time (hr)
n.s.
Realtive mCherry levels
hOPN4-mCherry
hOPN4dC-mCherry
F
NFAT-Luc
Blue light (475 nm)
Relative activity of NFAT-Luc
EC50=9.95 nW/mm2
Log (nW/mm2)
G
20 nW/mm2
violet 405 nm
blue 475 nm
green 520 nm
Relative Bioluminescence
Time (hr)

Figure S2. Characterization of OPN4dC in cell culture, related to Figure 1.
(A) Membrane localization of wild-type hOPN4-mCherry and hOPN4dC-mCherry in HEK293T cells. Indicated constructs were transfected to cells seeded on a cover glass in a 24-well plate. Cellular membrane and nuclei were stained by PlasMem Bright Green and DAPI, respectively. Scale bar: 20 µm.
(B) IP1 concentration in HEK293T cells expressing wild-type hOPN4-mCherry, hOPN4dC-mCherry and hM3Dq-mCherry. Cells expressing hOPN4s or mCherry control protein were exposed to blue LED light (1 µW/mm^2 for 20 or 30 min), followed by the IP-one assay (n = 4 replicates). Cells expressing hM3Dq or mCherry control were treated with 10 µM CNO for 20 min before the IP-one assay (n = 3 replicates). P value (*p < 0.05, ***p < 0.001) was calculated by Tukey's test. Solid bars and error bars present means with individual plots and SEM, respectively.
(C) Calcium response in HEK293T cells expressing hOPN4dC-mCherry. Cells expressing hOPN4s or mCherry as controls were treated with Fluo4-AM for at least 1 h before the recording. Fluorescence of Fluo4-AM was recorded every 2 s (Ex. 480 nm/Em. 519 nm, 500 ms expose time). The excitation light was also used for stimulating light of hOPN4s (n = 4 replicates). Solid circles and error bars present means and SEM.
(D) Calcium response in HEK293T cells expressing hOPN4dC-mCherry. Nano-lantern(Ca2+)_600 was expressed in the cells and substrate of nano-lantern (Endurazine) was added to the recording medium one hour before the recording. 475 nm blue light (300 ms, 5 times at an interval of 6 s) was exposed to cells (n = 4 replicates). P value was calculated by Two-way ANOVA. Solid circles and error bars present means and SEM.
(E) Left; NFAT-Luc activity in NIH3T3 cells expressing wild-type hOPN4-mCherry and hOPN4dC-mCherry was recorded constantly every 6 min. Twenty-four hours after the start of recording, blue LED light (1 µW/mm^2, 20 min) was illuminated to cells (n = 4 replicates). Right; fluorescence levels of mCherry in cells expressing wild-type hOPN4-mCherry or hOPN4dC-mCherry. P value was calculated by Two-way ANOVA. Solid circles and error bars present means and SEM.
(F) Effect of light intensity on the NFAT-Luc activation in 3T3 cells expressing hOPN4dC-mCherry. EC50 was calculated by sigmoid curve fitting of normalized Luc responses (n = 4 replicates). Solid circles and error bars present means and SEM.
(G) Effect of wavelengths of stimulation light on the NFAT-Luc activation in 3T3 cells expressing hOPN4dC. Blue (475 nm), green (520 nm) or violet (405 nm) LED light (20 nW/mm^2, 20 min) was illuminated to cells (n = 4 replicates for each condition).

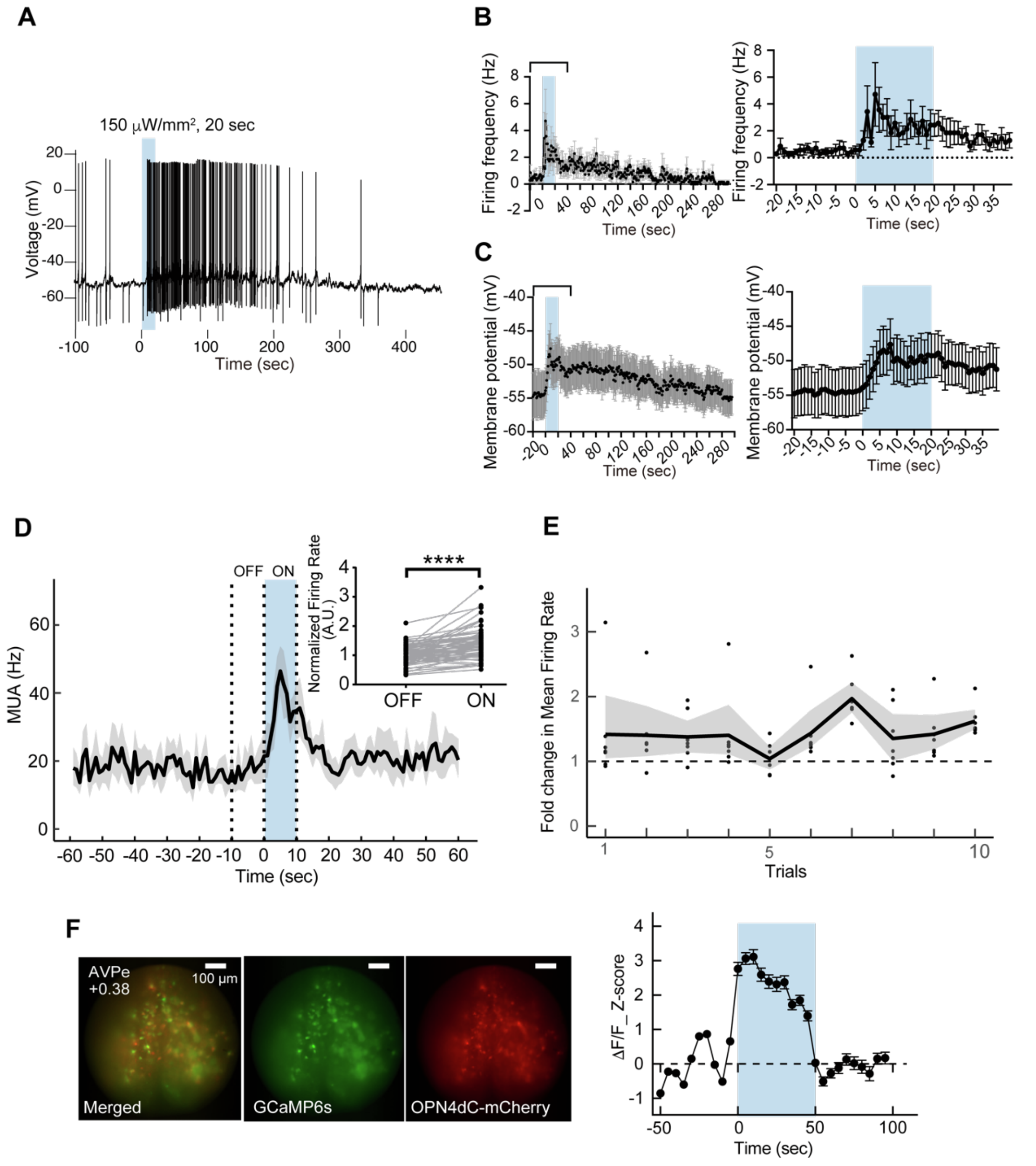

Figure S3. Response of hOPN4dC-expressing neurons to blue light, related to Figure 1.

(A) Representative trace of current-clamp recording from hOPN4dC-mCherry positive primary cortical neurons in culture. Recording was performed seven times using different neurons. Blue shading represents photo-stimulation.

(B) Firing frequency was plotted (n = 7 neurons from three independent preparations). Solid circles and error bars represent means and SEM. Magnified graph around the photo-stimulation period was shown in the right panel.

(C) Averaged membrane potential was plotted (n = 7 neurons from three independent preparation). Solid circles and error bars present means and SEM. Magnified graph around the photo-stimulation was shown in the right panel.

(D) Trace of multi-unit activity (MUA) from a representative electrode averaged across 10 light-stimulation trials (solid line, mean firing rates; gray shading, SEM) from *Calb1-Cre* mice in which hOPN4dC was expressed in *Calb1*-positive pyramidal neurons of the hippocampal CA1. Light stimulation (10 mW, 10 sec) was given non-invasively to neurons through the optic fiber positioned on the surface of the mouse brain. Individual plots of MUA data obtained from 7 electrodes show a significant increase during the light ON epochs (n=70 trials from 2 animals, right top inset). P value (****$p < 0.0001$) is from Wilcoxon matched-pairs signed rank test. Solid line and gray shading present mean and firing rates with individual plots.
(E) The fold changes of the firing rate in each trial were plotted. The firing rate during the 10-sec stimulation was normalized to the base value during the 10-sec pre-stimulation period (n= 7 electrodes from 2 animals).
(F) Calcium response of Q neurons expressing hOPN4dC-mCherry to light stimulation in the slice preparation. Left; representative fluorescence images of GCaMP6s and mCherry around the AVPe. Mixed AAV (AAV10-EF1α-DIO-hOPN4dC-mCherry and AAV10-hSyn-DIO-GCaMP6s) was bilaterally injected into the AVPe of *Qrfp-iCre* mice. Right; normalized fluorescence signals (ΔF/F) from GCaMP6s expressed in Q neurons was standardized, shown as ΔF/F_Z-score (n = 100 neurons from 2 animals). Solid circles and error bars present means and SEM. Scale bars: 100 μm.

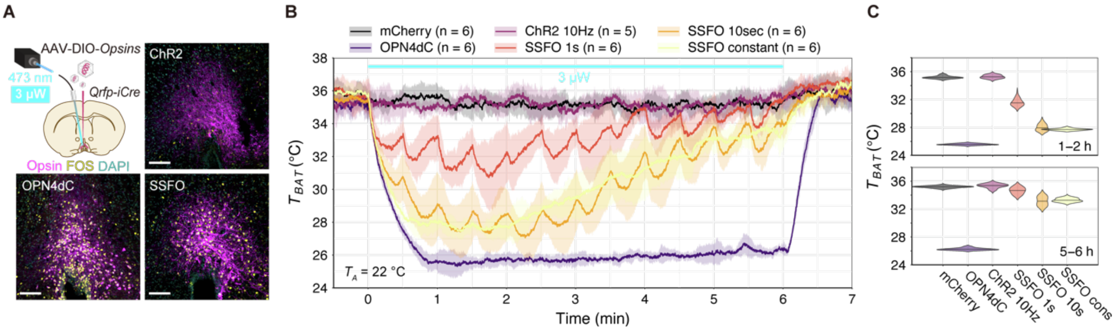

Figure S4. Optogenetic QIH induced by existing tools, related to Figure 2.

(A) Strategy for optogenetic excitation of Q neurons using AAV10-EF1α-DIO-ChR2(H134R), AAV10-EF1α-DIO-SSFO-EYFP, and AAV10-EF1α-DIO-hOPN4dC-mCherry to compare QIH induced by each opsin. Representative tissue images showing expression of opsins (magenta) and c-Fos (yellow) as well as nuclei stained with DAPI (cyan) in the AVPe 90 min after the start of photo-stimulation (473nm, 3 µW at the fiber tip, 100 µW mm^{-2}). hOPN4dC-mCherry was immunostained with anti-mCherry antibody. ChR2-EYFP and SSFO-EYFP were immunostained with anti-GFP antibody. Scale bars: 100 µm.

(B) Induction of hypothermia after hOPN4dC, ChR2 or SSFO-mediated photoactivation of Q neurons (n = 5–6 mice for each group). OPN4dC and control (mCherry) data are the same as the data shown in Figure 1B. Laser stimulation conditions for each group were: 3 µW, 10-ms-width, 10 Hz for 6 h (ChR2_10Hz); 3 µW, 1-s-width, every 30 min for 6 h, resulting in total 12 pulses (SSFO_1s); 3 µW,10-s-width, every 30 min for 6 h (SSFO_10s); 3 µW, constant for 6 h (SSFO_constant, mCherry and OPN4dC). Line and shading in are mean and SD, respectively.

(C) Violin plots of T_{BAT} at 1h–2h and 5h–6h after the beginning of laser stimulation.

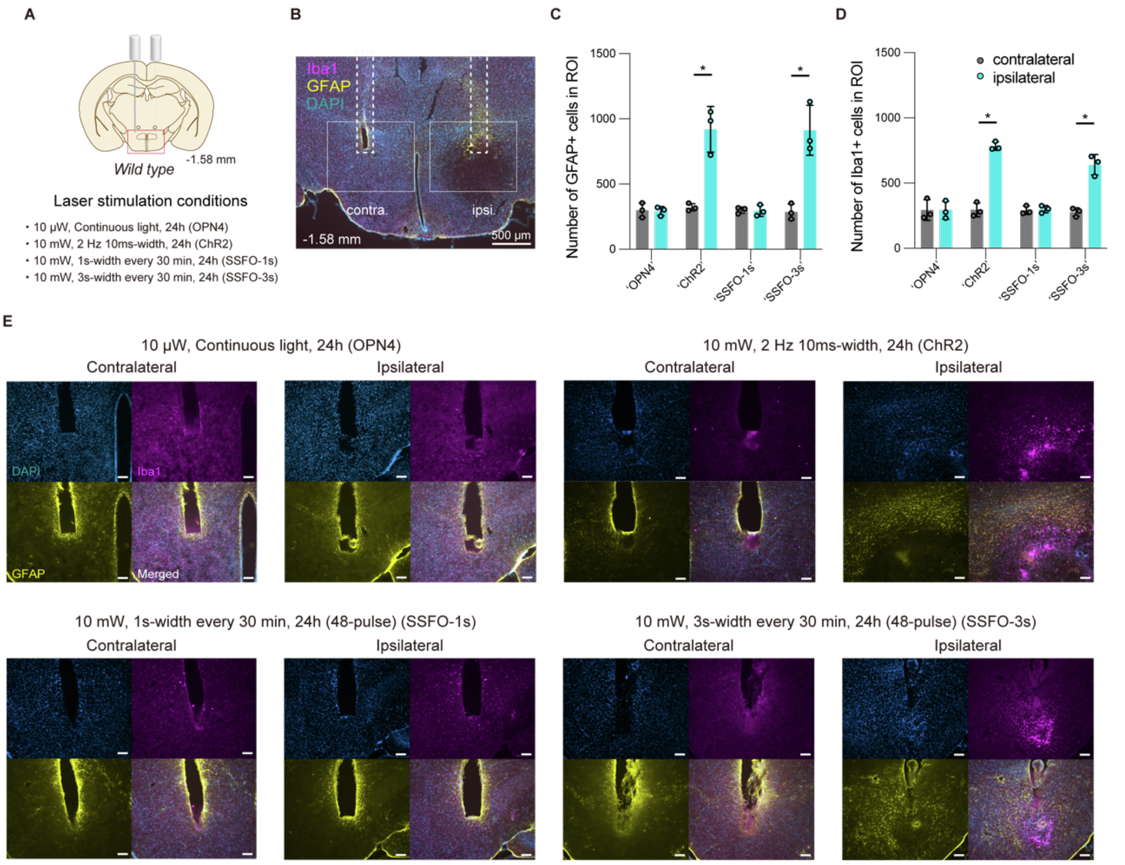

Figure S5. Activity of astrocyte and microglia in response to photo-stimulation through optic fiber, related to Figure 3.

(A) Schematic image of the hypothalamic area (red square) right underneath the laser stimulation site (AP - 1.58 mm from bregma). Optic fibers were bilaterally implanted above the lateral hypothalamus of wild-type mice (C57B6/J). The white squared area was used for quantifications of glial fibrillary acidic protein (GFAP) and ionized calcium-binding adaptor molecule 1 (Iba-1) immunoreactivity. 473 nm laser light was unilaterally delivered in appropriate conditions for ‘OPN4’ (10 µW, continuous), ‘ChR2’ (10 mW, 2 Hz, 10 ms-width), and ‘SSFO(1s or 3s)’ (10 mW, 1s- or 3-s width, every 30 min) for 24 h.

(B) Representative image depicting a coronal slice treated in ‘ChR2’ condition (10 mW, 2 Hz, 10 ms-width) exhibiting tissue lesioning on the ipsilateral side. Scale bar: 500 µm.

(C and D) Numbers of GFAP-positive astrocytes (C) and Iba-positive microglia (D) in ROI (1 mm square around fiber tip in the lateral hypothalamus) in the photo-stimulated (ipsilateral, blue) or non-photo-stimulated (contralateral, gray) side.

Animals were perfused 48 hours after the photo-stimulation. No glial accumulation was evident at ‘OPN4’ and ‘SSFO(1s)’ conditions (n = 3 mice; two-sided paired t-test, *$p < 0.05$). Data are means ± sem.

(E) Representative images with indicated side of photo-stimulation and laser condition. Scale bars: 100 µm.
